# Translational Pharmaceutical Perspectives on Quinones as Antioxidant and Antidiabetic Agents in Diabetes and Its Complications

**DOI:** 10.3390/pharmaceutics18091191

**Published:** 2026-09-20

**Authors:** Krishnendu Adhikary, Thirtsha Shohani Godwin, Adsaya Karunanithi, Riya Sarkar, Md. Abubakar, Sayoni Nag, Saurav Barman, Nilanjana Datta, Rajkumar Maiti

**Affiliations:** 1 Department of Medical Laboratory Technology, Paramedical College Durgapur, Durgapur 713212, West Bengal, India; 2 Research and Development Cell, Lovely Professional University, Pharwara 144411, Punjab, India; 3 Department of Pharmacy, Faculty of Allied Health Sciences, University of Jaffna, Jaffna 40000, Sri Lanka; 4 Department of Medical Laboratory Technology, Dr. B. C. Roy Academy of Professional Courses, Fuljhore, Durgapur 713206, West Bengal, India; 5 Department of Pharmacology and Toxicology, National Institute of Pharmaceutical Education and Research, Hajipur, Vaishali 844102, Bihar, India; 6 Department of Biotechnology, Brainware University, Barasat, Kolkata 700125, West Bengal, India; 7 Department of Soil Science, Centurion University of Technology and Management, Gajapati 761211, Odisha, India; 8 Department of Horticulture, Centurion University of Technology and Management, Gajapati 761211, Odisha, India; 9 Department of Physiology, Bankura Christian College, Bankura 722101, West Bengal, India

**Keywords:** Quinones, DM, oxidative stress, antidiabetic mechanisms, nanoformulation, drug delivery systems

## Abstract

**Background**: Diabetes mellitus (DM) is an extremely complex metabolic disorder, with many factors contributing to persistent hyperglycaemia, redox dysregulation, and micro- and macrovascular damage over time. There is growing evidence that oxidative stress is one of the main mechanisms of β-cell damage, insulin resistance, and consequent tissue damage, pointing to the need for therapies to modulate metabolic and redox responses. The extensive nature of quinones has revealed that they can be potential dual-acting redox-active compounds that may have antioxidant and antidiabetic activities. **Objectives**: This review aims to evaluate quinones from a pharmaceutical perspective, considering the chemical diversity and structure–activity relationships, the physicochemical parameters pertinent to drug development, and the mechanisms of action in terms of glucose homeostasis, insulin signalling, mitochondrial protection, and glycoxidative damage. **Methods**: Narrative review of the evidence from medicinal chemistry, experimental pharmacology, pharmacokinetics, formulation research, and clinical studies of the potential role of quinones in diabetes and its complications. The paper addresses the relationship between chemical structure and biological activity, redox-dependent mechanisms of action, tissue-protective actions, challenges associated with delivery, toxicity, and recent approaches to formulation. **Results**: Quinones influence several processes implicated in diabetes, including mitochondrial dysfunction, oxidative stress, pancreatic β-cell injury, insulin signalling impairment, AGE-RAGE activation, and chronic inflammation. Currently, CoQ10 has the strongest evidence in humans, while thymoquinone (TQ), pyrroloquinoline quinone (PQQ), anthraquinones, and mitochondria-targeted derivatives have mainly cellular and animal studies to support them. Low solubility, variable systemic exposure, rapid metabolism, and the potential for pro-oxidant effects at high concentrations continue to hamper therapeutic development of these compounds. **Conclusions**: Future research scopes will rely on rational derivatisation, precision delivery technologies, and clinically validated safety frameworks to translate redox pharmacology into effective therapeutic interventions.

## 1. Introduction

### 1.1. Global Burden of Diabetes Mellitus

The emergence of DM and its substantial clinical, economic, and social implications have made it one of the most pressing global public health issues of the 21st century. DM is characterised by growing metabolic dysfunction and multisystem repercussions, as well as by a chronic hyperglycaemic illness caused by insulin resistance or problems with insulin production [1]. An older population, more sedentary lifestyles, unhealthy diets, and more people living in metropolitan areas have all contributed to a dramatic increase in the disease’s prevalence in recent decades. Based on epidemiological projections, the global diabetes population is already in the hundreds of millions and is projected to grow in the following decades. Low- and middle-income nations, which lack the healthcare resources to handle the disease’s long-term effects, bear the brunt of the problem [2].

Persistent metabolic imbalance is the root cause of microvascular problems, including retinopathy, nephropathy, and neuropathy, as well as macrovascular disorders like coronary artery disease, stroke, and peripheral vascular disease. As a result of these issues, people’s health deteriorates, and more people die prematurely [3]. Treatment becomes much more complicated when patients with diabetes are at increased risk for complications such as persistent wounds, delayed tissue repair, and infections. Healthcare expenditures, both direct and indirect, as well as the costs of lost productivity, disability, and premature mortality, have a significant influence on the economy [4]. DM is a growing health problem due to its complex pathophysiology and rising prevalence; as a result, there is a pressing need for innovative ways to prevent and treat that address both the glycaemic control and the downstream pathogenic consequences of DM [5].

### 1.2. Oxidative Stress in DM Pathogenesis

When it comes to the causes and progression of diabetes, oxidative stress is an important molecular component. The imbalance between reactive oxygen species (ROS) generation and antioxidant defence mechanisms, which are responsible for neutralising these intermediates, is the cause of this condition [6]. Several interrelated mechanisms, including dysfunction of the mitochondrial electron transport chain, activation of protein kinase C, formation of advanced glycation end products, flux through the polyol pathway, and dysregulation of the hexosamine pathway, lead to increased ROS production in diabetes due to chronic hyperglycaemia [7].

Multiple biochemical pathways are activated when oxidative stress levels are too high, including lipid peroxidation, protein oxidation, and damage to nucleic acids [8]. The membrane integrity, signalling pathways, and mitochondrial function of insulin-sensitive organs are compromised by these cellular alterations, which lead to their death or functional decline [9]. The pancreatic β-cells are particularly vulnerable due to their inadequate natural capacity to defend themselves against ROS. Prolonged exposure to ROS impairs insulin secretion, disrupts glucose homeostasis, and contributes to pancreatic β-cell dysfunction [10].

Metabolic impairment and oxidative stress are two major causes of diabetic complications. Endothelial dysfunction caused by ROS in vascular tissues accelerates atherosclerosis and microvascular damage via inflammation, decreased nitric oxide bioavailability, and vascular remodelling [11]. Chronic inflammation is fuelled by oxidative stress, which activates redox-sensitive transcription factors such as NF-κB. This process, in turn, increases the production of cytokines and tissue damage. The interplay between hyperglycaemia, oxidative stress, and inflammation is a vicious cycle that speeds up the progression of illness [12]. Consequently, reducing oxidative stress has become a highly sought-after therapy strategy for improving diabetes outcomes and preventing its complications.

### 1.3. Quinones as Redox-Active Therapeutic Scaffolds

Quinones are a structurally diverse class of cyclic diketones that are well known for their unique redox-active properties and extensive pharmacological potential. Natural and synthetic quinones have been of significant scientific interest because of their ability to undergo reversible electron transfer reactions, thereby modulating cellular redox balance and affecting many biological pathways. This redox plasticity makes quinones particularly appealing as therapeutic scaffolds in diseases characterised by oxidative stress, inflammation, and metabolic dysfunction [13]. The biological activity of quinones is mainly defined by their involvement in one- and two-electron reduction reactions, leading to formation of semiquinone intermediates and redox cycling products. These mechanisms enable quinones to either promote controlled ROS generation for antimicrobial or anticancer effects, or to act as redox modulators that attenuate oxidative damage [14]. The compounds exhibited pharmacological versatility in various therapeutic areas such as antimicrobial, antiviral, anticancer, anti-inflammatory, and antioxidant action [15].

Importantly, quinone scaffolds can be chemically tuned for structural modulation to achieve target specificity, bioavailability, and safety profiles. Several compounds of clinical relevance, such as anthraquinones, benzoquinones, and naphthoquinones, have shown important biological activity against pathogenic microbes and inflammation processes. They are also useful therapeutically, as they inhibit microbial electron transport, inhibit biofilm formation, and modulate redox-sensitive signalling pathways. Oxidative imbalance plays a central role in chronic metabolic diseases, making quinones an attractive molecular scaffold for targeting multiple pathological processes through redox-based mechanisms [16].

### 1.4. Rationale for Quinones in Pharmaceutics

The multi-functional therapeutic potential and unique mechanistic versatility of quinones have led to increased interest in pharmaceutical research on these compounds. In the present era of drug development, there is a growing focus on developing molecular scaffolds that can target multiple interconnected pathological pathways simultaneously, particularly in complex disorders such as diabetes, where oxidative stress, chronic inflammation, infection, and impaired tissue repair coexist [17]. In this context, quinones are particularly advantageous due to their redox-active chemistry, which allows for the simultaneous modulation of several disease-driving processes [18].

**Figure 1:**
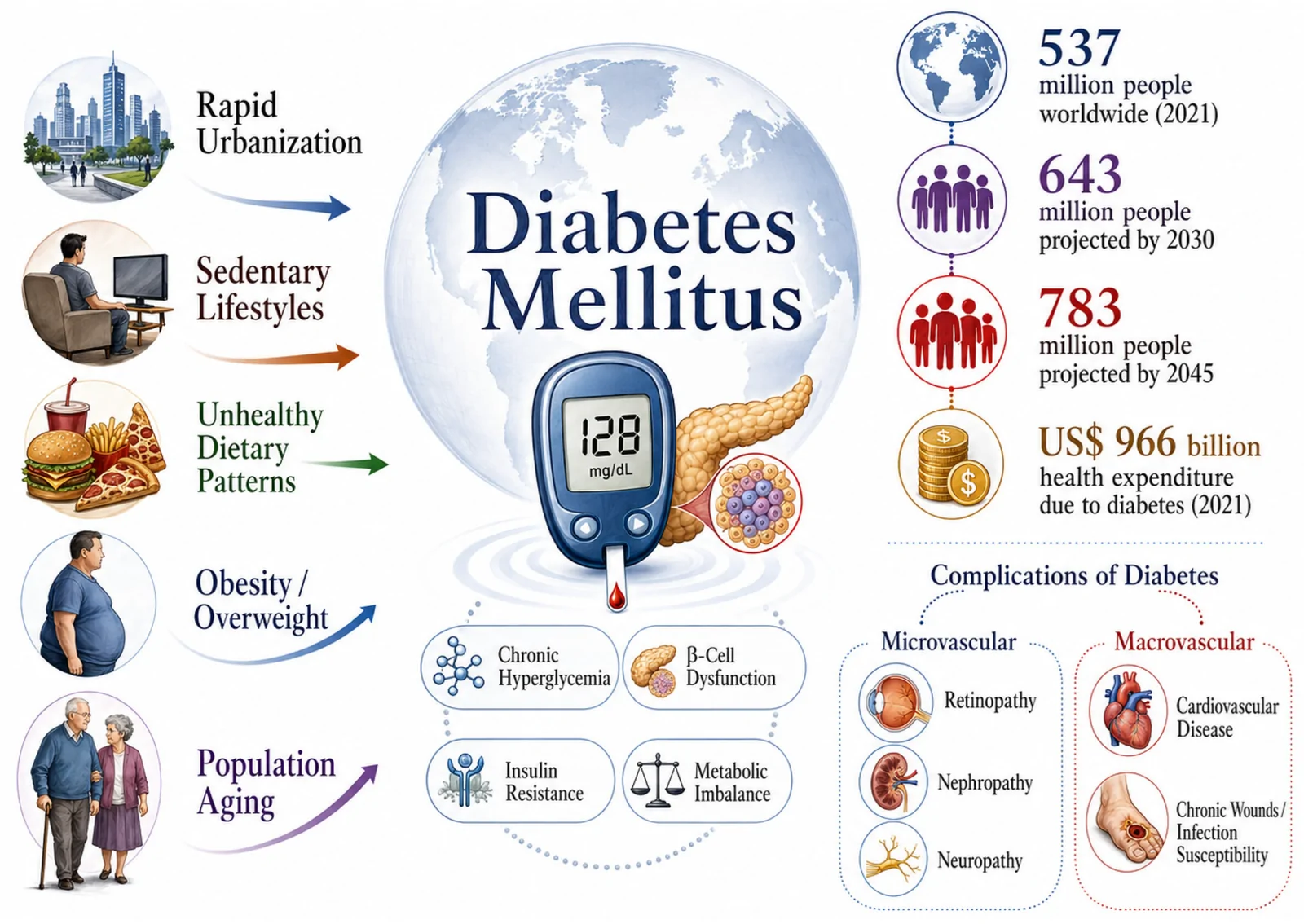
**Global burden and major drivers of DM.** This schematic illustrates how rapid urbanisation, sedentary lifestyle, unhealthy dietary patterns, obesity/overweight, and population ageing contribute to the rising prevalence of DM. The central metabolic axis highlights chronic hyperglycaemia, β-cell dysfunction, insulin resistance, and metabolic imbalance, which collectively promote microvascular complications such as retinopathy, nephropathy, and neuropathy, as well as macrovascular and systemic outcomes including cardiovascular disease, chronic wounds, and infection susceptibility. The figure also emphasises the increasing global disease burden, projected patient numbers, and associated healthcare expenditure. The scientific information presented in the figure was synthesised from the published literature [1,2,3,4,5,6].

Quinones have some attractive features from the pharmaceutical perspective. Their structurally flexible backbone allows extensive medicinal chemistry optimisation, resulting in better pharmacokinetic behaviour, improved selectivity for the target, and reduced off-target toxicity [14]. Quinones can also be incorporated in advanced drug delivery mechanisms like nanoparticles, hydrogels, liposomes, and polymeric scaffolds to improve solubility, controlled release, and site-specific delivery [19,20]. Formulations of this type are of special interest in the management of diabetic wounds where a localised sustained therapeutic action is required [21]. Figure 1 provides a schematic overview of the major epidemiological drivers, metabolic disturbances, and clinical consequences that together characterise DM as an emerging global health challenge.

Taken together, this review aims to critically examine the pharmaceutical potential of quinones in diabetes and its complications, with particular emphasis on their chemical diversity, redox-dependent mechanisms, structure–activity relationships, pharmacokinetic and formulation challenges, and the strength of available preclinical and clinical evidence. Particular attention is given to CoQ10 and other representative quinones to distinguish findings supported by human studies from those that remain at the experimental stage. By integrating pharmacological, pharmaceutical, and translational perspectives, this review seeks to identify the opportunities and limitations that should be considered when developing quinone-based interventions for diabetes.

## 2. Chemical Classification and Drug-Relevant Properties

### 2.1. Types of Quinones

Quinones are a family of natural and synthetic compounds that have the structure of an intramolecular unsaturated cyclic diketone, which can be quickly changed into other forms [22,23]. Natural quinones can be classified into three main categories: benzoquinone, naphthoquinone, and anthraquinone, which are made of one, two, and three ring structures, respectively, as shown in Table 1 [24].

Benzoquinone represents the basic building block of the quinone family, having two carbonyl groups on a benzene ring that can be found in plants, fungi, and bacteria [25,26]. It acts as the core structure for the synthesis of quinones and contributes to the biosynthesis of several bioactive compounds [27]. The relative positions of the two carbonyl groups on the benzene ring can create structural variations, which create two principal structural isomeric forms: ortho-benzoquinone (1,2-benzoquinone) and para-benzoquinone (1,4-benzoquinone) [23]. o-Benzoquinone is less stable than p-benzoquinone [28], and p-benzoquinone is a non-aromatic compound that is easily reduced to hydroquinone [26].

Structurally, naphthoquinones are naphthalene-derived molecules characterised by a benzene ring fused to a conjugated cyclic diketone, with the two carbonyl groups arranged in the para orientation, which is known as 1,4-naphthoquinone [29], and rarely in the ortho orientation, called 1,2-naphthoquinone. In plants, naphthoquinones typically occur in their reduced and glycosidic forms. In certain species, including Diospyros (Ebenaceae), they may also present as monomeric, dimeric, or even trimeric structures. Naphthoquinones are naturally coloured compounds, commonly responsible for brown or yellow colours, and they act as significant natural pigments [30]. As electrophiles, 1,4-naphthoquinones readily react with nucleophiles and undergo both 1,2-addition and 1,4-addition reactions. However, 1,4-dihydroxynaphthalenes are the reduced forms of 1,4-naphthoquinones and serve as nucleophiles. As a consequence, coupling reactions between the naphthoquinones and the 1,4-dihydroxynaphthalenes can yield dimeric products [31].

**Table 1:** Chemical classification of quinone and its structure and therapeutic properties.

Chemical Classification of Quinone	Representative Compounds	Structure	Structural Features	Natural Sources	Therapeutic Properties
Benzoquinone	CoQ10, idebenone, thymoquinone (TQ), embelin	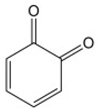 ortho-benzoquinone (1,2-benzoquinone)	Two carbonyl groups on a benzene ring	Least commonly found in nature. Mainly, this can be found in flowering plants, lichens, fungi, and several insects like millipedes and beetles. Carthamin, a dye from Carthamus tinctorius, is the most commonly investigated benzoquinone from a plant [26].	Antioxidant, anti-inflammatory, and neuroprotective effects
		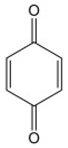 para-benzoquinone (1,4-benzoquinone)			Anti-inflammatory, anthelmintic, astringent, antipyretic, hypoglycaemic, antihyperlipidemic, antihypertensive, anti-diarrheal, antibiotic, contraceptive, painkiller, anti-erythrogenic, anticancer, and antioxidant properties
Naphthoquinone	β-Lapachone, lawsone, plumbagin, juglone, vitamin K derivatives	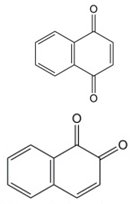 1,4-naphthoquinone 1,2-naphthoquinone	Two carbonyl groups on Naphthalene ring	Can be found in plants, such as Verbenaceae and Bignoniaceae families, microorganisms, some animals, including starfish, sea stars, and sea urchins, and particular fungi types [26].	Antitumour, antileishmanial, antiproliferative, antimalarial, antiviral, antibacterial, and antifungal activities
Anthraquinone	Emodin, rhein, aloe-emodin, chrysophanol, alaternin	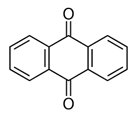	Two carbonyl groups on tricyclic anthracene core	Mostly found in the families such as Liliaceae, Rhamnaceae, Fabaceae, Coelospermum, Scrophulariaceae, Polygonaceae, and Rubiaceae [32]	Antimicrobial, anticancer, anti-inflammatory activities

### 2.2. Plant-Derived Bioactives

Juglone (5-hydroxy-1,4-naphthoquinone), a phenolic compound found in walnuts, exhibits antimicrobial, antifungal, antioxidant, anticancer, anti-angiogenic, and skin-related activities [33]. Its hydroxyl and keto groups form an intramolecular hydrogen bond that contributes to radical stabilisation and biological activity, while structural modifications influence its therapeutic properties [34]. Juglone is also a potent redox cycler capable of reacting with oxygen and reactive species [35]. Traditionally, fresh juice from unripe black walnut (*Juglans nigra*) hulls has been used against topical fungal infections, with juglone considered a major active compound [36]. It also shows antibacterial activity, particularly against Gram-positive *Staphylococcus aureus*, through redox cycling [33,37].

Plumbagin is a significant naphthoquinone that shows several therapeutic activities, such as antioxidant, antidiabetic, anti-inflammatory, antifungal, antibacterial, analgesic, antileishmanicidal, anti-atherosclerosis, and potent anticancer properties [38]. Plumbagin, a vitamin K_3_ analogue with strong pro-oxidant properties [39], exhibits broad-spectrum anticancer activity through multiple molecular mechanisms against several types of cancers, such as breast cancer, brain tumour, cholangiocarcinoma, leukemia, esophageal cancer, canine cancer, hepatocellular carcinoma, melanoma, lung cancer, osteocarcinoma, squamous carcinoma, and prostate cancer [38]. Plumbagin, mainly obtained from the roots of *Plumbago zeylanica* L., displays potential activity against hormone-refractory prostate cancer [40]. Mainly, it modulates several major pathways involved in cancer progression, including inflammatory responses, apoptotic signalling, ROS-mediated pathways, cell cycle regulation, and major signalling cascades like PI3K/AKT/mTOR and STAT3/PLK1/AKT [41]. Furthermore, it acts as an anticoagulant, as it resembles the structure of vitamin K, which might help in regulating cardiovascular diseases [42]. A previous study showed that plumbagin has poor water solubility and limited gastrointestinal absorption; it is safe and displays weak antimalarial activity [43].

Lawsone, also known as 2-hydroxy-1,4-naphthoquinone, is mainly found in *Lawsonia inermis* and displays many biological activities, such as antioxidant and anticancer properties, as well as anti-inflammatory, antibacterial, and antifungal activities [44]. Based on previous studies, it has shown potent antifungal activity against *F. oxysporum*, *A. flavus*, *A. niger*, and *Penicillium* sp. [45]. Lawsone is considered one of the safest antioxidants due to its natural origin, with high efficacy [44]. Furthermore, it can reduce the proliferation of colon cancer cells by suppressing NF-κB activity, thereby reducing the expression of cyclin B1 and cdk1 [46]. It displays potential anticancer properties through inhibiting cell proliferation and inducing apoptosis [47]. Moreover, the leaf extract of *Lawsonia inermis* shows potent antidiabetic activity due to increased GLUT-4 and stimulates pancreatic β-cell regeneration [48]. Previous studies show that lawsone has significant wound -healing properties when applied topically [49]. In vitro findings revealed that lawsone modulated the NF-κB and TNF-α pathways, with outcomes similar to those of prednisolone [50].

### 2.3. Therapeutically Relevant Quinones

The pharmacological activity of a quinone does not necessarily make it suitable for diabetes therapy. Candidate compounds must be evaluated according to metabolic relevance, attainable exposure, safety, formulation viability, and the robustness of evidence. Coenzyme Q10 (CoQ10) is the most clinically developed, whereas PQQ, thymoquinone, emodin, rhein, plumbagin, and lawsone remain supported mainly by preclinical evidence [51,52,53,54,55,56,57,58]. CoQ10 (ubiquinone) is an endogenous benzoquinone with ten isoprene units that supports mitochondrial energy production and antioxidant defence [51,52,53]. Although obtained through endogenous synthesis and diet, its availability may be insufficient under certain conditions. CoQ10 has been investigated in metabolic, cardiovascular, neurological, and mitochondrial disorders [52,54]. However, its hydrophobicity and relatively large molecular size limit oral bioavailability, prompting development of analogues with modified quinone cores or hydrophobic tails to improve pharmacological properties [18]. MitoQ and idebenone similarly illustrate quinone modification for mitochondrial targeting, although their diabetes-specific clinical value remains uncertain. Menadione, anthracyclines, and highly reactive endogenous quinones are better regarded as examples of redox pharmacology and toxicity than as chronic diabetes therapies [51,52,53,54,55,56,57,58].

Mitoquinone, a dietary supplement, is the synthetic analogue of ubiquinone, which is used as a potent superoxide scavenger that reduces lipid peroxidation [55]. Furthermore, it is structurally modified to accumulate within mitochondria, enhancing its potent antioxidant properties while managing the functional integrity of the mitochondria. This characteristic makes this agent a promising candidate for treating a wide range of oxidative stress-based diseases, such as metabolic disorders, cardiovascular diseases, liver diseases, and neurodegenerative diseases [56]. Mitoquinone is not FDA-approved for a specific disease, but several clinical trials have been done with it as an investigational drug. Especially, previous studies indicated that this can act as post-exposure prophylaxis against SARS-CoV-2 [57].

Thymoquinone is traditionally used to treat several diseases, such as diabetes, asthma, and bactericidal infections. It can be obtained from the seeds of *Nigella sativa*. It demonstrates many pharmacological outputs, such as antioxidant, anticancer, neuroprotective, antimicrobial, radioprotective, hepatoprotective, anti-inflammatory effects in mice, and immunomodulatory properties, cardio-protective effect, and anti-metabolic syndrome in rats. These properties have attracted researchers to focus more on thymoquinones [58,59]. Thymoquinone exhibits potent antioxidant properties through the formation of cytoprotective enzymes, which prevent cell damage due to oxidative stress [60]. In vivo studies show that oral administration of TQ at a dose of 100 mg/kg for one week considerably enhances the total antioxidant levels in mice [61]. Moreover, it shows anticancer properties through several mechanisms, including enhancing the activity of antioxidant enzymes to decrease cancer cell progression, protecting cytochrome P450 enzymes, deactivating the NF-κB signalling pathway through modulation of cytokine production, and controlling oncogene expression and normal cell protection during cancer treatment [58].

In addition, 9,10-anthraquinones are one of the important quinone nutraceuticals that have a planar three-ring aromatic system with two ketone groups at the 9 and 10 positions. Anthraquinones and their derivatives exhibit potent pharmacological properties such as antimicrobial, anti-inflammatory, anticancer, antiplatelet, laxative, and neuroprotective effects. Moreover, previous studies have shown better activity against malaria infection and multiple sclerosis. Clinically, anthracycline, an anthraquinone derivative, has been used as an anticancer drug [62].

Menadione has demonstrated broad in vitro anticancer activity across several cancer types, including mammary, hepatic, breast, oral, haematological, and bladder cancer cells. Moreover, it remains effective against parental leukemia cell lines and multidrug-resistant leukemia cell lines [63]. The primary mechanism of cytotoxicity is mainly driven by oxidative stress generated through quinone redox cycling, resulting in the elevation of ROS levels and ultimately causing DNA damage and subsequent cell death [64]. Phase I clinical investigations have reported that menadione has acceptable tolerability and synergistic cytotoxic effects when combined with agents such as vitamin C and mitomycin C [65].

### 2.4. Structure–Activity Relationships

For over a century, quinones and their derivatives have been investigated because of their chemical and electronic properties. Especially, their redox potential feature allows them one- and two-electron reductions, leading to semiquinone and hydroquinone formation, respectively. Even though quinones display several reactive and bioactive properties, further structural modifications can enhance their effectiveness [30]. The presence of some key functional substituents, such as cyano and nitro groups, and their position, can significantly alter the bioactivity profile of the compound [66].

The α-glucosidase and protein tyrosine phosphatase 1B (PTP1B) inhibitory activities can be substantially enhanced by increasing the number of hydroxyl groups in the core structure. For instance, 2-hydroxy-3-methyl-anthraquinone displays weak α-glucosidase inhibitory activity, while polyhydroxylated quinones, such as emodin, chrysophanol, and alaternin, show significant potent action [67]. The study conducted by H. A. Jung, M. Y. Ali, and J. S. Choi reveals that the presence of free hydroxyl groups at the C-1, C-6, and C-8 positions appears to be essential for the antidiabetic activity. Notably, alaternin carries an additional hydroxyl group at the C-2 position, demonstrating threefold higher PTP1B inhibitory activity than emodin, confirming the key role of hydroxylation at the C-2 position in profoundly enhancing antidiabetic activity. Moreover, the antidiabetic activity of emodin was higher than aloe-emodin, confirming the importance of the methyl substituent at the C-3 position, while aloe-emodin contains a hydroxymethyl group at the C-3 position [68].

A previous study reveals that the antioxidant property of CoQ10 and its analogues can be enhanced by adding morpholine and piperazine at the C-6 position instead of adding a hydroxyalkyl or alkoxy substituent [69]. Moreover, the antioxidant activity increases along with the decrease in the side chain length [31]. The previous study conducted by Y. Guo et al. showed that the antiviral property of anthraquinones is significantly influenced by both their location and the number of phenolic hydroxyl groups in their structures. Moreover, the antiviral activity can be enhanced by the presence of a keto-phenol arrangement within the same benzene ring. In addition, the addition of hydroxyl groups on the same benzene ring and the number of keto-phenol systems have been shown to further improve inhibitory potency. The second and sixth positions of the anthraquinone ring showed a significant HIV-1 nucleocapsid-inhibiting property [70].

The SAR of quinones reports a systematic framework on how structural variations can impact the mechanism of action, efficacy, and potency of biological properties. This comprehensive study on SAR provides the foundation for designing more potent quinone-based anticancer agents with an improved therapeutic profile. The efficacy of the anticancer property can be influenced by the types of substituents, such as cyano and nitro groups, and their positions on the ring. Incorporating electron-withdrawing substituents such as cyano and nitro groups typically enhances the property by facilitating the transfer of electrons or stabilising the quinone radical. However, electron-donating substituents such as methoxy and amino may suppress the formation of radicals and diminish the activity. Furthermore, the anticancer property can be significantly enhanced by ortho- and meta-substitutions, as they are closer to the reactive centres that participate in ROS generation and redox cycling. The expansion of the core structure of quinone through ring fusion can profoundly alter the bioactivity through improving its redox characteristics, planarity, and conjugation. For instance, anthraquinone and naphthoquinone with larger ring systems often display greater activity. Furthermore, the suitable linkers and conjugation can enhance the pharmacokinetic property of the agent, such as its stability, solubility, and absorption [19,71]. The biological activity of a quinone is influenced by more than the number of rings in its structure. The number and position of hydroxyl groups, the length of the side chain, lipophilicity, electron distribution, and electrophilic reactivity all affect target binding, membrane passage, and redox behaviour. Hydroxyl groups at specific locations on anthraquinones can enhance binding to α-glucosidase and PTP1B, while substitution of a methyl group with a hydroxymethyl group can affect binding to both enzymes and cellular uptake. In CoQ10-related compounds, shortening or modifying the hydrophobic side chain can increase polarity and improve access to the mitochondria. These modifications must nevertheless be approached carefully because increasing electrophilic or redox activity may also promote nonspecific protein binding and ROS formation. Candidate selection should therefore balance metabolic activity, tissue exposure, and safety rather than focusing only on antioxidant potency [31,66,67,68,69,70,71,72].

### 2.5. Physicochemical and Drug-Likeness Properties

Pharmacokinetic and pharmacodynamic profiles, as well as drug safety, are usually governed by physicochemical and molecular characteristics of the molecule. In modern drug discovery, there is an increasing emphasis on strategies to enhance ADME properties with target affinity. Moreover, the idea of “drug-likeness” has been developed to evaluate fundamental molecular properties, such as molecular weight and lipophilicity, which always influence the bioavailability of a molecule [73].

Quinones are one of the major naturally occurring organic compounds found broadly in nature. Historically, these compounds were highly utilised for several purposes before their structural and functional properties were scientifically recognised. Characteristically, quinones are observed as yellow crystals with a chlorine-like irritating odour. They are poorly soluble in alcohol, water, ether, alkalis, and hot petroleum ether. Functionally, they act as a strong oxidising agents and are readily reduced to hydroquinone. Quinones are often utilised as chemical indicators due to their noticeable changes in physical appearance, such as colour shifts, during chemical reactions. Therefore, this can be employed in dye-related industrial applications [74]. The framework of quinones is characterised by the presence of C=C bonds, conjugated C=C bonds, a carbonyl group, and C=O bonds. Consequently, they are involved in several chemical reactions, such as reduction reactions, 1,4-addition reactions of a conjugated double bond, C=C bond addition reactions, and carbonyl addition reactions. Moreover, their chemical properties are strongly influenced by the position and nature of the substituent group on the quinone or on a neighbouring ring [75]. Quinones show many resemblances to α, β-unsaturated ketones. They mainly exhibit redox behaviour due to electrophilic carbonyl groups and their capability to interact with nucleophiles through polarised double bonds. As a potent reactive organic molecule, quinones interact with biological systems, exerting several pharmacological activities and toxicities. Usually, quinones interact with biological complexes or enzymes through covalent or non-covalent binding. Their polarity profile significantly plays a major role in cellular uptake and membrane permeability, allowing them to reach their potential targets and subsequent binding. More lipophilic quinones may tend to stay longer in the lipid layer. In addition, structural factors, such as chirality, also impact the bioactivity [76]. For instance, “Sennidin A” demonstrates greater stimulation of glucose incorporation compared to “Sennidin B” [77]. Therefore, the overall biological profile of quinones and their metabolism, including pharmacological and toxicological outcomes, is determined by the physicochemical properties of quinones [76].

**Figure 2:**
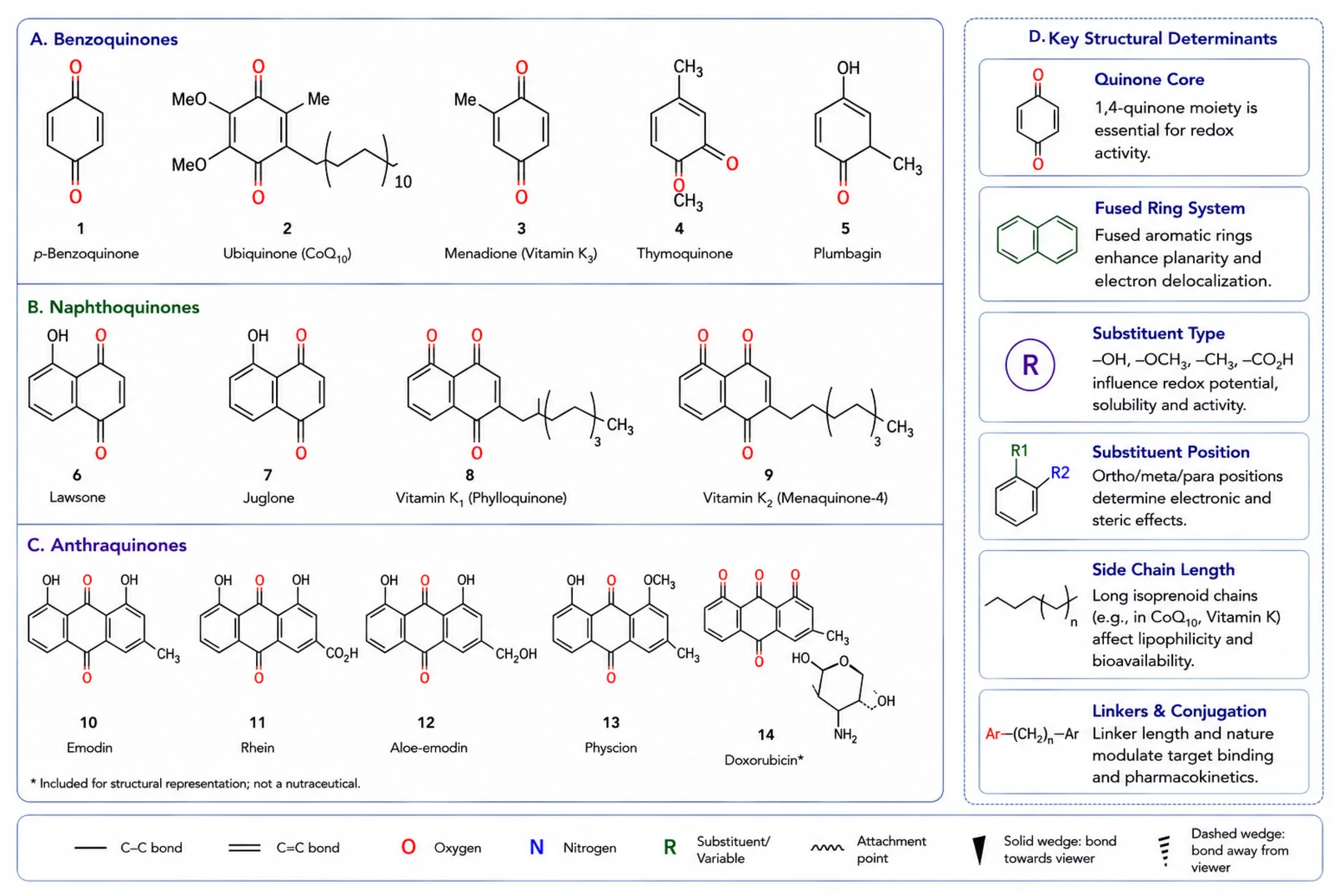
**Representative quinone scaffolds and major structure–activity relationship determinants.** The figure illustrates the chemically important quinone derivatives and their structural features. Panels (**A**–**C**) show synthetic quinone-analogues like CoQ10/idebenone-like derivatives, mitochondria-targeted quinone systems, and multitarget quinone hybrids. (**D**) Representative quinone structures and key structural determinants, highlighting the major quinone classes and the functional groups that influence their chemical and biological properties. Together, these structural features offer a chemical basis for tuning redox behaviour, target interaction, bioavailability, and therapeutic efficacy of quinone-based compounds. The scientific information presented in the figure was synthesised from the published literature [22,23,24,25,26,27,28,29,30,31,32,51,54,58,62,63,67,68,76,78].

In biological systems, quinones commonly act as a major electron transport agent via reversible redox mechanisms. They exhibit a broad range of therapeutic properties, such as antibacterial, antiviral, antifungal, antimalarial, and antitumour effects, establishing them as a valuable “privileged structure” in drug discovery. A privileged structure refers to a specific molecular framework that has the ability to bind or interact with multiple biological targets, thereby demonstrating a broad range of therapeutic activities. The structural modifications generate selective and potent ligands for diverse biological targets. Moreover, these modified structures generally show favourable drug-like properties, which can be further used as the starting point for the development of novel bioactive compounds and the design of compound libraries [78]. For instance, the study conducted by Ratchanok Pingaew et al. developed a novel 1,4-naphthoquinone-derived sulfonamide substituted with 6,7-dimethoxy groups that showed potent antimalarial activity [79]. In 2016, the study conducted by Eduardo H.G. da Cruz et al. developed selenium-containing quinone-derived triazoles with two redox centres that showed their potent antitumour activities [80]. This privileged structure led to the acceleration of the development of new potential drug candidates, which can be used across several therapeutic areas [78]. In an effort to complement the mechanistic discussion, representative quinone-based structures and structure–activity relationship features are illustrated in Figure 2, emphasising how redox-active cores, fused ring systems, substitution positioning, and linker modifications influence their pharmacological potential.

## 3. Antioxidant Mechanisms

Numerous physiological functions in the human body, including respiration, digestion, and metabolism, may produce ROS and reactive nitrogen species (RNS), which are often free radicals or have the ability to produce free radicals. However, their excessive formation causes oxidative damage to protein, lipid, and DNA, and disrupts the cell signalling [81].

### 3.1. Redox Cycling and Radical Scavenging

Highly reactive atoms, molecules, or ions with unpaired electrons that react intensely with other compounds are known as free radicals. ROS, RNS, and reactive sulfur species (RSS) are molecules of oxygen, nitrogen, and sulfur that are the main sources of these radicals [82]. The excessive generation of ROS causes harm to human health, resulting in several disease conditions, such as diabetes, cancer, and cardiovascular disease [83]. Usually, the antioxidant agents, especially radical scavenging agents, scavenge peroxide radicals, resulting in the termination of radical chain reactions [84]. Several scientific studies have demonstrated that natural antioxidants from natural sources are one of the significant factors in preventing many diseases [83].

Natural anthraquinones, such as purpurin, emodin, aloe-emodin, chrysophanol, rhein, and physcion, show potent antioxidant properties through O_2_^•−^ radical scavenging [83,85]. Moreover, vitamin K-hydroquinone, the reduced form of vitamin K, showed potent radical scavenging property compared to α-tocopherol and ubiquinol [86]. Furthermore, a previous study showed that naphthoquinone also exhibits radical scavenging property [87]. CoQ10 shows potent antioxidant activity via scavenging the free radicals, and thus prevents DNA damage [88]. In addition, plastoquinone and ubiquinone can prevent lipid peroxidation, DNA damage, and protein oxidation by scavenging free radicals in plants. However, they demonstrate their antioxidant activity in their reduced forms, plastoquinol and ubiquinol. Especially, plastoquinol scavenges the singlet oxygen very effectively [89]. Juglone, or 5-hydroxy-1,4-naphtoquinone, a natural compound, contains a structure made of an intramolecular hydrogen bond between keto groups and hydroxyl groups. This bond can donate the hydrogen atom, which can directly affect the stability of free radicals. Juglone can act as an antioxidant as well as a pro-oxidant based on its concentration [33]. Moreover, a study conducted on the antioxidant activity of plumbagin showed that it has a potent radical scavenging property due to its active hydrogen in the phenolic hydroxyl group in its structure [90]. Quinones should not be described as antioxidants under all biological conditions. Their effects depend largely on how they are reduced inside the cell. NQO1 and related enzymes often catalyse two-electron reduction to produce relatively stable hydro-quinone forms that can scavenge radicals and interrupt lipid-peroxidation reactions. The semiquinone intermediates are formed by one-electron reduction, which can transfer electrons to molecular oxygen, generate superoxide, and regenerate the original quinone. Thus, the protective or damaging effect of a quinone on the cell is determined by its concentration, subcellular location, availability of oxygen, and reductase activity, and the strength of the surrounding antioxidant system. Such a mechanism can explain the fact that a compound can be protective at a lower exposure and pro-oxidant and cytotoxic at a higher dose. This concentration-dependent redox window is of particular relevance for the long-term application of quinones in diabetes [14].

### 3.2. Antioxidant Enzyme Modulation

Major antioxidant enzymes are those that may either directly prevent the production of free radicals or indirectly boost other endogenous antioxidant defences. Superoxide dismutase (SOD), catalase (CAT), and glutathione peroxidase (GPx) are the main antioxidant enzymes. A key component of cellular antioxidant processes is the Gpx. It uses glutathione (GSH) to catalyse the detoxification of lipid peroxides and H_2_O_2_ in the cytosol and mitochondria. By inhibiting lipid oxidation, it shields cells from oxidative damage.

During these reactions, GSH is oxidised to glutathione disulfide (GSSG). Then the GSSG is reduced back to GSH by glutathione reductase (GR) using NADPH and maintains the cellular redox balance [81]. CAT is a tetrameric heme protein predominantly found in peroxisomes of aerobic cells. It acts as an antioxidant defencive mechanism by catalysing the dismutation of hydrogen peroxide into molecular oxygen and water. Alterations in CAT activity are closely associated with various disorders [81,91]. SOD is another powerful intracellular antioxidant enzyme crucial for cellular protection from oxidative damage. This enzyme is involved in the dismutation of superoxide anions (O_2_^•−^) into molecular oxygen (O_2_) and hydrogen peroxide (H_2_O_2_). However, metallic cofactors, such as Zn, Cu, Fe, and Mn, are required for their effective activity, which varies based on the isoform with specific subcellular localisation. For instance, the cytosolic Cu/Zn-containing SOD (SDO1) is predominantly found in the cytosol, the mitochondrial Mn-containing SOD (SDO2) is present in mitochondria, and the extracellular Cu/Zn-containing SOD (SOD3) is found in plasma. However, SOD supplementation is essential for immune system protection, as its level declines with age [81,91].

Lawsone is a naphthoquinone obtained from henna leaf extract [92]. It shows potent antioxidant property through antioxidant enzyme modulation. It can form hydrogen bonds between the hydroxyl group and a carbonyl group with SOD. Moreover, it can generate a hydrophobic interaction with SOD [93]. Lipopolysaccharide-induced sepsis can impair the antioxidant defence system in our body. However, based on the recent study, aloe-emodin can reverse the reduction in SOD and the activity of GSH-Px in lipopolysaccharide-induced sepsis [94].

### 3.3. Inhibition of Glycoxidative Damage

The advanced glycoxidation end products (AGEs) and lipoxidation end products (ALEs) are extensively researched for their oxidative and glycoxidative damage capability, and are considered as vital products in the progression of diabetic complications, such as microangiopathy, retinopathy, chronic kidney disease, and other pathological conditions [95]. Several approaches have been identified to inhibit the formation of AGE/ALE by targeting different inducers, including ROS, metal ions, and intermediate products such as reactive carbonyl species. Therefore, the usage of antioxidants, reactive carbonyl compound quenchers, and ion chelators is considered the most suitable strategy to inhibit the formation of AGE/ALE. However, in natural or synthetic compounds, the inhibition of AGE/ALE formation may act through multiple mechanisms, involving combinations of antioxidant, RCS quenching, and metal ion chelation [96]. Based on a previous study, potential inhibitory activities of the main anthraquinones in *A. vera* were studied. Aloe-emodin, emodin, and chrysophanol showed effective inhibition of the formation of AGE in a BSA-Glc model system. Fructosamine, a reactive carbonyl intermediate, including glyoxal and methylglyoxal, can enhance AGE formation. However, emodin has potent inhibition for the formation of glyoxal and fructosamine, and thus inhibits the formation of AGE. Therefore, emodin is considered a potent antiglycation component in *A. vera* [97]. Moreover, rhein, an anthraquinone compound, and vitamin C can hinder the accumulation of AGEs [98,99].

### 3.4. Nrf2/Keap1 Activation

In the cellular defence mechanism, the Keap1-Nrf2 pathway plays a crucial role in exogenous and endogenous stresses, which are generated from ROS and electrophiles [100]. This activation is engaged in the control of the transcription of numerous genes that regulate antioxidants, which permit cellular homeostasis, and detoxification genes, which are involved in the removal of toxins and carcinogens, thereby reducing cellular damage [101]. In the regulatory regions of target genes, the transcription factor Nrf2 binds with Maf proteins to the antioxidant response element (ARE), while Keap1, a repressor protein, binds to Nrf2 and suppresses it by promoting its degradation through the ubiquitin proteasome pathway [100]. PQQ shows the ability to activate Nrf2 and transcriptional activation of TFB1M, TFB2M, and Tfam, which are connected with the PGC-1α pathway activation [102]. Chrysophanol, a natural anthraquinone found in rhubarb, shows potent antioxidant activity through activating the Keap1/Nrf2 pathway [103]. Furthermore, emodin can induce the antioxidant defence mechanism in several ways, including regulating the antioxidant enzymes and molecules responsible for Nrf2/Keap1 activation [104]. Aloe-emodin reduces oxidative stress by activating the Nrf2 pathway [105].

### 3.5. Mitochondrial Redox Targeting

**Figure 3:**
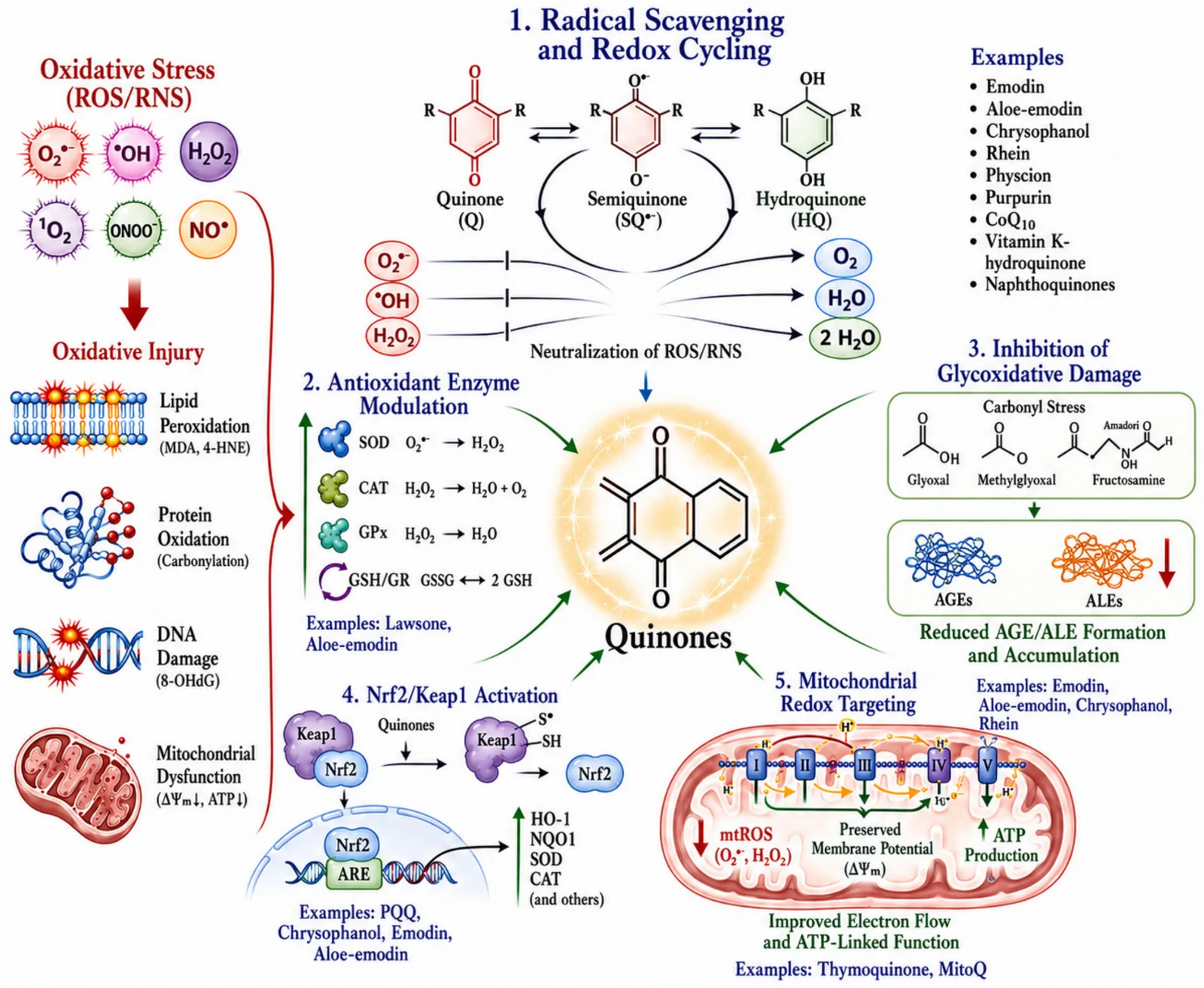
**Antioxidant mechanisms of quinones in diabetes-associated oxidative stress.** This schematic illustrates the multifunctional antioxidant actions of quinones against ROS/RNS-mediated cellular injury. Quinones participate in reversible redox cycling between quinone, semiquinone, and hydroquinone states, enabling radical scavenging and restoration of redox balance. In addition, they enhance endogenous antioxidant defences through modulation of SOD, CAT, GPx, and GSH/GR systems; inhibit glycoxidative damage through reduction of carbonyl stress, AGE/ALE formation, and molecular injury; activate the Keap1/Nrf2/ARE axis to upregulate cytoprotective antioxidant genes; and maintain mitochondrial electron flow, membrane potential, and ATP-linked function. Together, these mechanisms lower oxidative burden, preserve lipids, proteins, and DNA; sustain cellular signalling; and enhance cell survival under diabetic conditions. The scientific information shown in the figure was synthesised from the published literature [81,82,83,84,85,86,87,88,89,90,91,92,93,94,95,96,97,98,99,100,101,102,103,104,105,106,107,108].

The excessive generation of ROS causes oxidative damage to cellular macromolecules. Generally, mitochondria form mitochondrial ROS (mROS) during strong oxidative phosphorylation [106]. These are known as 106 mitochondrial-targeted antioxidants. TQ is one of the significant antioxidants that targets mitochondria. It can directly act on ROS because of the oxidation of TQ [107]. MitoQ (mitoquinone) is a quinone-derived compound that acts as an antioxidant targeting mitochondria. It is different from conventional antioxidants due to its easy entrance into the mitochondrial interior. It has a lipophilic triphenylphosphonium cation (TPP), which is covalently bound to the CoQ_10_, resulting in rapid entry and potent radical-scavenging properties [108]. The antioxidant role of quinones is schematically represented in Figure 3, highlighting how these redox-active scaffolds counter oxidative injury through radical neutralisation, antioxidant enzyme modulation, glycoxidative stress suppression, Nrf2/Keap1 activation, and mitochondrial redox protection.

## 4. Anti-Diabetic Mechanism of Quinones

Quinones exert their antidiabetic actions through several related metabolic pathways rather than a single molecular target. Different compounds have been reported to preserve pancreatic β-cell function, support mitochondrial energy production, activate AMPK and PI3K/Akt signalling, promote GLUT4 translocation, delay carbohydrate digestion, and limit AGE–RAGE-mediated inflammation. This range of actions may be relevant to diabetes because defective insulin secretion, peripheral insulin resistance, and oxidative tissue injury usually develop together. However, the evidence is not equally strong for every mechanism. Enzyme inhibition, glucose uptake, and intracellular signalling have been investigated mainly in biochemical assays, cultured cells, and animal models. Human evidence is considerably more developed for CoQ10 than for other quinones. The proposed effects on the gut microbiota are even less certain and should remain described as preliminary until they are confirmed through controlled microbiome and metabolomic studies [109,110,111,112,113,114,115,116,117,118,119,120,121,122,123,124,125,126,127,128,129,130,131,132,133,134,135,136,137,138,139,140,141,142,143,144,145,146,147,148,149,150,151,152,153,154,155,156,157,158,159,160,161,162,163,164,165,166,167,168,169,170,171,172,173,174,175,176].

### 4.1. Regulation of Insulin Secretion and Pancreatic β-Cell Protection

Quinones play a significant role in protecting pancreatic β-cells, primarily through their antioxidant and mitochondrial-supporting properties. Pancreatic cells are vulnerable to oxidative stress. Excessive oxidative stress contributes to mitochondrial dysfunction, impaired insulin secretion, and progressive β-cell apoptosis. Quinones interact with pancreatic beta cells through their redox properties due to their conformational flexibility and their ability to participate in electron transfer reactions, modulating oxidative stress and mitochondrial function [109].

Quinones inhibit lipid peroxidation of mitochondrial membranes by maintaining membrane stability [110]. A previous in vivo study demonstrates that CoQ10 shows protective effects on pancreatic β-cells against mitochondrial stress induced by staurosporine using the mouse pancreatic β-cell line MIN6 [111]. Chronic metabolic stress can impair insulin secretion and further damage β-cells and lead to apoptosis of β-cells [112]. Quercetin upregulates antioxidant defences via stimulation of Sirtuin 3 (Sirt3), a mitochondrial deacetylase that amplifies resistance to oxidative stress; this condition arises when there are lower levels of apoptotic markers [113]. Quinone derivatives may modulate inflammatory signalling pathways and enhance cellular redox balance, consequently assisting in the maintenance of β-cell mass and function. Quinones inhibit NF-κB activation [114], minimising cytokine-mediated β-cell dysfunction by downregulating pro-inflammatory pathways [115,116]. They stimulate the nuclear factor erythroid 2-related factor 2 (NRF2) signalling pathway via their electrophilic interactions with cysteine residues in Kelch-like ECH-associated protein 1 (KEAP1) [114].

Quinones help upregulate these enzymes and reduce ROS levels, thereby contributing to the maintenance of β-cell mass and function. An in vivo study showed that CoQ10 reversed renal SOD activity and hepatic glutathione peroxidase activity, and oxidised glutathione concentration in the brain, in streptozotocin-induced diabetic rats [117]. An oral formulation of mitoquinone (MitoQ) is safe and effective in human phase 2 studies, as a mitochondria-targeted antioxidant protects the survival and function of pancreatic β-cell lines under glucotoxic and glucolipotoxic conditions [118]. TQ ameliorated the impairment of glucose-stimulated insulin secretion due to chronic exposure of β-cells to glucose overload. This protection is associated with regulating chronic accumulation of malonyl-CoA and elevation of acetyl-CoA carboxylase [119]. Therefore, quinones are considered a promising therapeutic agent for conserving the integrity of pancreatic beta cells.

### 4.2. Glucose Uptake Enhancement

Among the key metabolic defects in diabetes is mitigated glucose uptake in peripheral tissues and impaired glycolysis. In DM, insulin resistance, oxidative stress, mitochondrial dysfunction, and chronic inflammation disrupt these pathways. Quinones aid in the enhancement of glucose uptake by the activation of crucial signalling pathways and their redox properties [115]. Evidence that quinones improve insulin-mediated glucose uptake and glycolysis through the stimulation of AMP-activated protein kinase (AMPK), phosphatidylinositol-3 kinase/protein kinase B (PI3K/Akt), GLUT4 translocation, antioxidant defence, and mitochondrial bioenergy. β-Lapachone (BL), a renowned substrate of NAD(P)H:quinone oxidoreductase (NQO1), has been proven to stimulate AMPK activation via NQO1 activation [120]. Tanshinone IIA, which belongs to the quinone family, activates AMPK and increases glucose uptake, promotes mitochondrial biogenesis, and boosts ATP production [121,122]. Plumbagin improved high glucose-induced cell apoptosis and insulin resistance of HTR8/SVneo through activation of the AKT/mTOR pathway, hence enhancing GLUT4 translocation and glucose uptake [123]. Sennidin A, an anthraquinone derivative, stimulates glucose incorporation in a phosphatidylinositol 3-kinase (PI3K)- and Akt-dependent, but IR/IRS1-independent manner, in rat adipocytes [124]. Rhinacanthin C, a major bioactive naphthoquinone that helps in GLUT4 and GLUT2 translocations and increases glucose uptake [125]. PQQ directly impacts the metabolism of glucose via pathways such as the Entner–Doudoroff pathway and indirectly via ERK1/2 and mTOR. It enhances conversion of glucose into gluconic acid, therefore indirectly impacting glucose uptake and storage [126,127].

### 4.3. Modulation of Insulin Signalling Pathways (PI3K/Akt, AMPK)

Under normal physiological conditions, after a meal, insulin instantly binds to its receptor [128,129]. It leads to autophosphorylation and activation of the ligand-receptor complex and triggers downstream signalling through this pathway, which plays a crucial role in glucose uptake, glycogen synthesis, and lipid metabolism. Activation of PI3K produces phosphatidylinositol-3,4,5-triphosphate (PIP3), which activates Akt, eventually boosting the GLUT4 transporter and its translocation to the cell membrane, which increases glucose utilisation [130]. In diabetes, this pathway is defective because of insulin resistance, leading to impaired glucose uptake.

According to the previous literature, certain quinones show insulin-mimicking action, which involves activation of insulin receptors and enhances the downstream signalling of the phosphoinositide 3-kinase/protein kinase B (PI3K/Akt) pathway and the AMPK pathway. For example, demethylasterriquinone B1 (DAQ B1) triggers the insulin receptor tyrosine kinase, leading to autophosphorylation and activation of the PI3K/AKT pathway [131]. Another study showed that emodin improved insulin sensitivity through upregulating the expression of IRS-1, PI3K, and Akt-ser473 stimulated phosphorylation in type 2 diabetic KKAy mice models [132]. TQ, a novel CoQ10 derivative, and embelin have been documented to improve insulin sensitivity by stimulating PI3K/Akt phosphorylation and reducing insulin resistance [133,134,135]. Likewise, quinones have an impact on the AMPK pathway. AMPK is a paramount regulator of cellular energy homeostasis, glucose homeostasis, and insulin sensitivity [136]. In diabetes, the AMPK-SIRT1-FOXO pathway plays a crucial role in preserving glucose homeostasis. Under conditions of chronic oxidative stress, intracellular energy levels shift, increasing the AMP/ATP ratio [137]. This activates AMPK. In skeletal muscle, activation of AMPK improves glucose absorption by activating AS160 and driving GLUT4 translocation to the plasma membrane [138]. In tandem in the liver, AMPK inhibits glycogen synthase and activates glycogen phosphorylase, ensuring a consistent flow of glucose during energy stress [139], thus lowering blood glucose levels under insulin-resistant conditions. Meanwhile, FOXO1 controls gluconeogenesis in the liver by activating genes such as PEPCK and G6Pase, ensuring glucose production under low-energy conditions [140]. Even more, SIRT1 is another energy regulator and promotes insulin secretion from the pancreas. It enhances insulin sensitivity by deacetylating IRS proteins, reducing inhibitory serine phosphorylation, and enhancing tyrosine phosphorylation [141]. This process enhances the downstream insulin signalling, which collectively enhances insulin secretion. Additionally, AMPK activation alleviates oxidative stress, inflammation, and lipotoxicity, thereby alleviating diabetes-induced organ damage [136]. Research shows that emodin enhances GLUT4 translocation and glucose uptake into the myotube in an AMPK and ROS-calmodulin-dependent protein kinase kinase (CaMKK)-dependent manner [142]. Coenzyme Q10 maintains mitochondrial integrity [143]. Tanshinone IIA, which belongs to the quinone family, activates AMPK and enhances insulin sensitivity [121,144]. Moreover, quinones stimulate the phosphorylation of essential intermediates such as insulin receptor substrate (IRS), which commences the signalling cascade necessary for insulin-dependent glucose uptake.

### 4.4. Digestive Enzyme Inhibition

α-Amylase and α-glucosidase are carbohydrate-digesting enzymes [144]. The control of postprandial blood glucose levels is one of the key strategies in managing DM [145]. Several natural quinones, such as benzoquinones, anthraquinones [146], and naphthoquinone derivatives [147,148], have shown potent inhibitory activity against both α-glucosidase and α-amylase enzymes. Embelin is a naturally occurring para-benzoquinone that possesses potent inhibitory activity on α-glucosidase [149]. Emodin and its derivatives, such as aloe-emodin, alaternin, and questin, showed significant inhibitory activity against α-glucosidase, with IC50 values of 1.02 μM for emodin, 1.40 μM for aloe-emodin, 0.99 μM for alaternin, and 136.19 μM for questin. It has been proven that the number of hydroxyl groups had an impact on α-glucosidase inhibition, particularly with the hydroxyl group at the R1 position being important [150]. Therefore, quinones show enzyme inhibitory activity through their non-covalent binding to biological complexes [115].

### 4.5. Advanced Glycation End-Product (AGE) Suppression

Advanced glycation end-products (AGEs) are chemically diverse compounds formed through non-enzymatic reactions between reducing sugars and proteins, nucleic acids, and lipids. These reactions generate unstable Schiff bases, which rearrange into Amadori products and subsequently form AGE precursors and stable AGEs [151,152]. AGEs may be endogenous or exogenous and can occur as short- or long-lived proteins and peptides [153,154,155]. Persistent AGE accumulation, promoted by hyperglycaemia, contributes to diabetic pathology through extracellular matrix cross-linking and activation of the RAGE pathway. AGE–RAGE signalling impairs GLUT4 translocation, promotes insulin resistance, stimulates NADPH oxidase-dependent ROS production, and activates ERK/MAPK, JAK/STAT, AP-1, and NF-κB pathways, thereby contributing to oxidative stress, inflammation, and chronic diabetic complications [152,155,156,157].

Aloe-emodin, rhein, and emodin, which are effective components of Guanxin Xiaoban capsules, could strongly inhibit the advanced glycation end product–receptor for advanced glycation end product (AGE-RAGE) signalling pathway [115]. TQ, a quinone derived from *Nigella sativa* (black seed), through its antioxidant potential, may reduce the production of methylglyoxal-derived Hb-AGE formation [158]. In a previous study, a Mediterranean diet supplemented with coenzyme Q10 modulated the postprandial metabolism of advanced glycation end products, reduced AGE levels, and increased AGER1 and GloxI mRNA levels in elderly men and women [159]. PQQ has a potential role as an antioxidant to reduce oxidative damage during hyperglycaemia by AGE inhibition in the cells of the human hepatocellular liver carcinoma (HepG2) [160]. Ethanolic extract of *Lawsonia inermis* and its known constituent lawsone showed significant inhibition of AGE formation and exhibited 77.95%, and 79.10% inhibition at concentrations of 1500 µg/mL and 1000 µg/mL [161]. A previous study reported that several natural dietary anthraquinone derivatives, including aloin, aloe-emodin, chrysophanol, emodin, physcion, and rhein, were evaluated for their anti-glycation effects [162]. The anthraquinones at 100 µM reduced fructose-, methylglyoxal-, and glyoxal-induced human serum albumin glycation by 21% (aloe-emodin) to 92% (aloin), 17% (physcion) to 94% (emodin), and 16% (anthraquinone) to 67% (emodin), respectively [162]. Hence, quinones can prevent AGE products due to their antioxidant, metal-chelating [163], carbonyl-trapping, and antiglycation properties.

### 4.6. Gut Microbiota Modulation

In healthy individuals, gut microbiota contribute to metabolism, nutrient absorption, and immune regulation. Microbial imbalance, or dysbiosis, involving reduced beneficial or increased pathogenic bacteria, is associated with diabetes [164,165,166]. Dysbiosis may increase gut permeability, allowing antigens to enter circulation and promote β-cell damage and hormonal disturbances [167,168]. Bacterial lipopolysaccharides can trigger inflammation and impair insulin signalling, contributing to insulin resistance [169]. Beneficial microbiota produce short-chain fatty acids (SCFAs), including butyrate, propionate, and acetate, which improve insulin sensitivity, stimulate GLP-1 secretion, and enhance glucose uptake [169,170,171,172]. Reduced SCFAs and increased branched-chain amino acids may further aggravate insulin resistance in type 2 diabetes [173,174].

**Figure 4:**
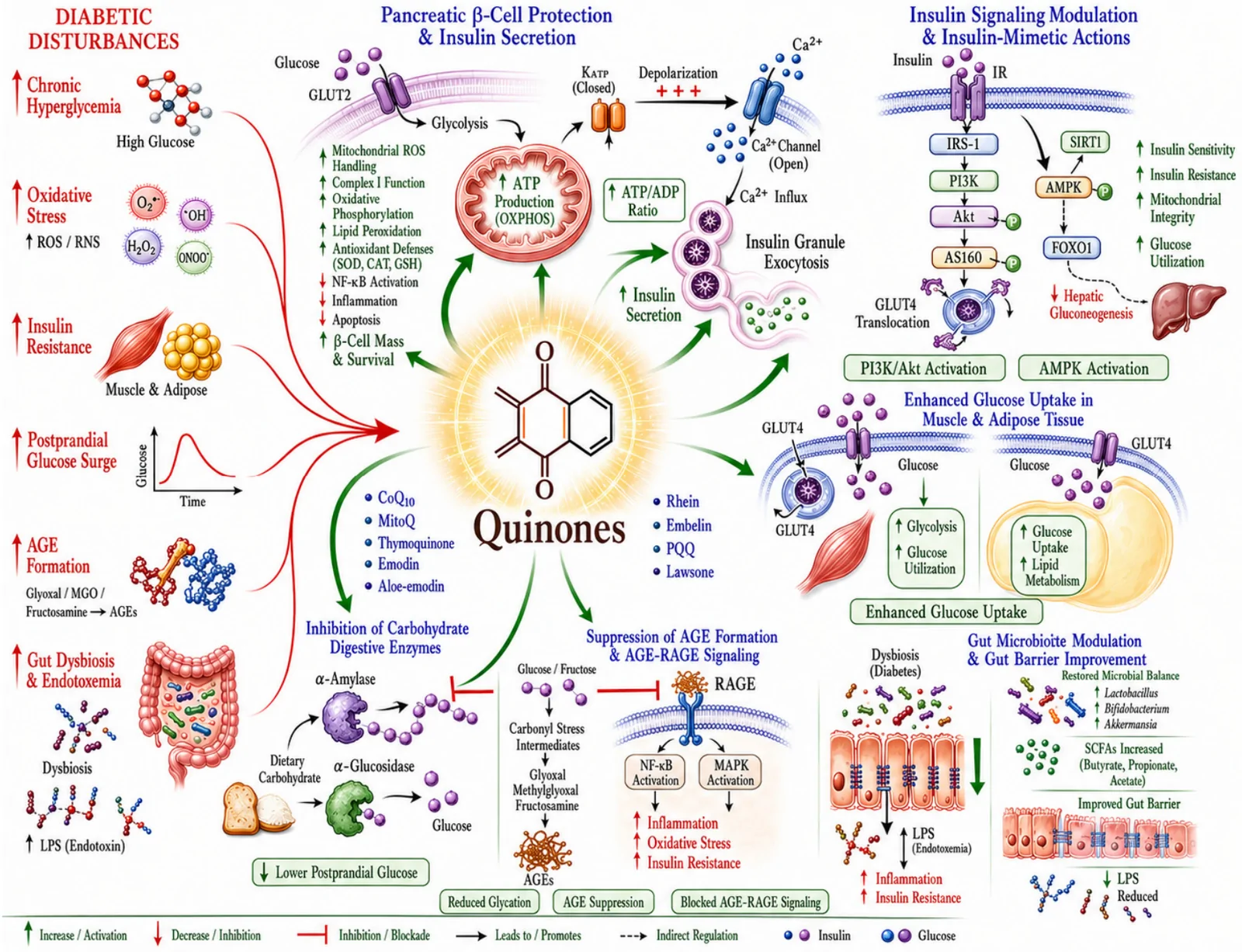
**Mechanistic overview of the antidiabetic actions of quinones.** Here is how quinones hit the multiple, interconnected defects that drive the pathophysiology of diabetes. Quinones improve mitochondrial function in pancreatic β-cells and protect them from oxidative and inflammatory injury, preserve β-cell mass, and enhance insulin secretion. They also enhance insulin signalling via the PI3K/Akt and AMPK-related pathways, facilitate GLUT4 translocation, and boost glucose uptake and utilisation in muscle and adipose tissues. Simultaneously, quinones are also effective in reducing postprandial hyperglycaemia by inhibiting α-amylase and α-glucosidase, inhibiting AGE formation and AGE–RAGE signalling, and alleviating downstream inflammatory and oxidative stress responses. The figure further highlights their role in modulating gut dysbiosis, reducing endotoxemia, and improving barrier integrity, thereby reinforcing metabolic control through gut–metabolism crosstalk. Collectively, these mechanisms position quinones as multifunctional scaffolds with strong potential for diabetes management. The scientific information presented in the figure was synthesised from the published literature [109,110,111,112,113,114,115,116,117,118,119,120,121,122,123,124,125,126,127,128,129,130,131,132,133,134,135,136,137,138,139,140,141,142,143,144,145,146,147,148,149,150,151,152,153,154,155,156,157,158,159,160,161,162,163,164,165,166,167,168,169,170,171,172,173,174,175,176].

Quinones are believed to influence the modulation of gut microbiota in DM by acting as microbial growth factors, metabolic regulators, and redox mediators. They facilitate microbial balance by supporting anaerobic respiration as electron carriers, and numerous intestinal microbes are unable to produce adequate quinones by themselves; therefore, they depend on neighbouring microbes or are supplied through the diet for quinones. In the intestine, this cross-feeding chain enhances the colonisation and ecological balance of gut microbiota by supporting helpful bacteria. Therefore, they mitigate inflammation and enhance insulin sensitivity. Menaquinones are needed as growth-promoting factors for human gut bacteria [175]. Therefore, quinones contribute to the maintenance microbial diversity. The dietary vitamin K quantity was altered by intestinal flora and affected the flora composition in male and female C57BL6 mice [176]. Aloe-emodin, emodin, and rhein, as effective components of Guanxin Xiaoban capsules, could effectively prevent the advanced glycation end product–receptor complex (AGE-RAGE) in the signalling pathway, and this mechanism is indicated to be associated with changes in intestinal flora [115]. Although individual quinones act through distinct molecular targets, their antidiabetic effects converge on a limited number of metabolic control nodes linking β-cell survival, insulin responsiveness, postprandial glucose handling, and glycoxidative stress (Figure 4, Table 2).

## 5. Quinones in the Management of Diabetic Complications

Persistent hyperglycaemia leads to microvascular and macrovascular complications such as nephropathy, retinopathy, neuropathy (microvascular), and cardiovascular complications (macrovascular), which are largely driven by excessive formation of ROS [177], activation of inflammatory pathways [178], production of advanced glycation end-products [179], mitochondrial dysfunction [180], and endothelial damage [181]. Therefore, therapeutic agents like quinones that can regulate oxidative stress and mitochondrial homeostasis are of great interest [127].

### 5.1. Diabetic Nephropathy: Mitochondrial Protection and ROS Regulation

Diabetic nephropathy is a major complication of diabetes, characterised by mesangial expansion, glomerulosclerosis, GBM thickening, albuminuria, tubulointerstitial fibrosis, and progressive renal dysfunction [182]. Mitochondrial dysfunction and excessive ROS production are important contributors to its pathogenesis [183,184]. Diabetes-associated metabolic disturbances can impair mitochondrial respiratory-chain enzymes, increasing electron leakage and ROS generation, particularly at Complexes I and III [185,186,187]. Under normal conditions, mitochondrial SOD2 converts superoxide to H_2_O_2_ and helps maintain redox balance. Excessive mitochondrial ROS can damage proteins, lipids, and DNA; impair electron transport chain efficiency; and further aggravate mitochondrial dysfunction [185,188].

Partially reduced semiquinones, in contrast, are reactive chemical species that can cause cell death by alkylation interactions with DNA, proteins, and lipids [189]. They mitigate oxidative stress by preserving the mitochondria and ROS regulation. Deficiency in mitochondrial-oxidised primary CoQ10 may constitute a potential precipitating factor for diabetic nephropathy. As a consequence, CoQ10 supplementation may be renoprotective in type 2 diabetes via preservation of mitochondrial function [190,191]. Human kidney 2 (HK-2) cells are protected from oxidative stress-induced damage caused by high glucose concentrations by PQQ, a naturally occurring redox-active quinone molecule. PQQ counteracts renal fibrosis by mitigating mitochondrial dysfunction, reducing ROS production, and inhibiting the activation of the NF-κB/pyroptosis pathway under conditions of DN and hyperglycaemia [127]. NAD(P)H:quinone oxidoreductase 1 (NQO1), the largest multimeric enzyme complex of the mitochondrial respiratory chain, protects cells from oxidative stress and damage caused by toxic quinones [192,193]. Therefore, it could be a novel target for diabetes-induced nephropathy.

### 5.2. Diabetic Neuropathy: Neuroprotective and Anti-Inflammatory Actions

One of the most typical chronic complications is diabetic peripheral neuropathy (DPN), which often emerges as a distal, symmetric, sensorimotor neuropathy [194]. The numerous factors pertaining to chronically unregulated hyperglycaemia contribute to the development of DPN, for instance, the polyol pathway, advanced glycation end-product formation, oxidative stress, and activation of protein kinase C [195,196]. DPN is a major contributor to sensory, motor, and autonomic dysfunction and has the potential to cause ulcers, limb amputation, and death [197]. A previous study indicated that TQ provides a protective benefit on peripheral nerves in DPN rats. The mechanism may be mediated by modulation of the inflammatory reaction. The study showed that TQ decreased COX-2, IL-1β, IL-6, and caspase-3 expression in the sciatic nerve or in isolated cultured Schwann cells. It also ameliorated the inhibition of Schwann cell proliferation and protected against Schwann cell apoptosis, enhanced nerve conduction velocity, and reduced DPN-induced morphological changes and demyelination of the sciatic nerve [198]. According to a previous study in 2012, repeated oral administration of PQQ (20 and 40 mg/kg, once a day for 4 weeks, starting on day 1 after injury) diminished both thermal and mechanical hyperalgesia and also reduced muscle atrophy. The mechanisms underlying the anti-hyperalgesic activity of PQQ are the reduction of pro-inflammatory mediators such as tumour necrosis factor alpha (TNF-α) and lipid peroxide malondialdehyde (MDA) levels in rats with chronic constriction injury (CCI) of the sciatic nerve [199]. The CoQ10 supplement serves as an adjuvant therapy to gabapentin for patients with diabetic neuropathy, improving glycaemic control and clinical symptoms by reducing inflammation and oxidative stress [200]. According to an observational study in a streptozotocin-induced chick embryo diabetic neuropathy model, vitamin K1 reduced oxidative damage by blocking the formation of ROS or modulating antioxidant enzymes, and acted as an anti-inflammatory agent by suppressing nuclear factor κB (NF-κB) signal transduction. These findings indicate that vitamin K1 could play a significant role in managing oxidative stress and inflammation associated with diabetic neuropathy [201]. The existing literature shows that quinones serve as therapeutic agents in diabetes-induced neuropathy through their neuroprotective effects and reduce inflammation.

### 5.3. Diabetic Retinopathy: Vascular Endothelial Protection

Diabetic retinopathy (DR), a major cause of preventable blindness, is driven by persistent hyperglycaemia, oxidative stress, endothelial dysfunction, inflammation, and vascular injury [202,203]. Excessive retinal ROS damages proteins and DNA and promotes inflammatory signalling, leading to endothelial injury, pericyte loss, basement membrane thickening, and macular edema. Concurrent neuronal apoptosis, glutamate toxicity, and reduced neuroprotective factors further contribute to retinal structural and functional impairment [204].

Quinones have unique biological aspects in protecting vascular endothelial cells. A core mechanism is mitochondrial stabilisation by reducing the mitochondrial membrane potential, inhibiting the formation of ROS, and aiding in ATP synthesis in retinal neural cells under diabetic conditions [205]. A previous investigation showed that the topical application of CoQ10 eyedrops in conjunction with vitamin E d-α-tocopheryl poly 1000 succinate ameliorated the functional and structural changes of a range of retinal neural cells and matrix metallopeptidase-9 (MMP-9) levels on DR-induced neurodegeneration using a type 2 diabetes mouse model, and could be considered as a complementary treatment against DR [206]. Prior scholarly work has established that two novel mitoprotective short-chain quinones have shown better protection of retinal pathology against oxidative damage, reactive gliosis, retinal ganglion cell loss, retinal thinning, and vascular leakage [202].

Moreover, quinones affect redox-sensitive transcription pathways, which are essential to maintaining homeostasis of vascular endothelium. A primary mechanism is the activation of the nuclear factor erythroid 2-related factor 2 (Nrf2) antioxidant defence signalling pathway through the upregulation of redox-sensitive endogenous antioxidant genes, including NAD(P)H: NQO1 and heme oxygenase-1 (HO-1), which further diminish ROS accumulation, inflammation, and endothelial cell apoptosis induced by hyperglycaemia in retinal cells [207].

Lastly, quinones protect the vascular endothelium by exhibiting anti-inflammatory and anti-angiogenic properties. Quinones (CoQ10) decrease ROS-induced activation of NF-kB and pro-inflammatory cytokines by suppressing the expression of the NF-kB gene and lowering the synthesis of pro-inflammatory cytokines, so they reduce leukocyte adhesion and inflammation of vascular endothelial cells [208]. Both are causes of vascular damage in DR. Furthermore, increasing vascular endothelial growth factor (VEGF) is a major pathological change in diabetic retinopathy [209], which causes neovascularisation and vascular leakages in the retina [210]. Aloe-emodin is a derivative of anthraquinone, which reduces the level of VEGF [211,212].

### 5.4. Cardiovascular Complications: Modulation of Oxidative and Inflammatory Pathways

Diabetic cardiomyopathy is characterised by myocardial hypertrophy, fibrosis, and cardiac dysfunction, and contributes substantially to diabetes-related mortality [213]. Hyperglycaemia promotes mitochondrial dysfunction, ROS generation, oxidative stress, AGE accumulation, and impaired vascular signalling [214,215]. Excessive ROS activates NF-κB, promoting inflammatory mediators and contributing to hypertrophy, fibrosis, and cardiomyocyte injury [216,217]. ROS-dependent activation of the NLRP3 inflammasome further induces caspase-1, IL-1β, and IL-18 release, aggravating inflammation and insulin resistance [218]. AGE-related TLR4 activation also stimulates NF-κB and MAPK pathways, promoting inflammation, fibrosis, and cardiac cell death [219,220]. Conversely, Nrf2 counteracts oxidative stress and suppresses NF-κB-mediated injury [221,222]. Several quinones have been identified as potent cardiac protective agents. A few examples of these quinones are cryptotanshinone, purpurin, embelin, tanshinone, aloe-emodin, aloin, and plumbagin. They reduce inflammation by regulating the NLRP3 inflammasome and limiting the production and activation of Ang II-induced NLRP3 inflammasome [223].

According to a previous study, CoQ10 supplementation may help in reduce oxidative stress by neutralising ROS [224,225], activating the Nrf2/ARE pathway [208], and lowering oxidative damage to cells and tissues. It is linked to protection against endothelial dysfunction and myocardial damage [226], and has the ability to protect against atherogenesis through NO-related pathways in human umbilical vein endothelial cells, all of which contribute to its potential cardioprotective benefits in diabetic complications.

PQQ exhibits potential positive effects in health, such as anti-oxidative, anti-inflammatory, and anti-diabetic effects [227] and cardioprotective effects [228]. Prior investigations have demonstrated that PQQ diminished blood glucose levels [229] and lessened myocardial hypertrophy and myocardial fibrosis in a rat model of diabetes through inhibiting the activation of the NF-κB/NLRP3 inflammasome signalling pathway, improving the decrease in mitochondrial membrane potential while maintaining mitochondrial function and reducing ROS production [230].

### 5.5. Wound Healing and Skin Complications in Diabetes

Diabetic foot ulcers and impaired wound healing are among the major complications of diabetes [231]. Normal wound healing progresses through haemostasis, inflammation, proliferation, and remodelling, but diabetes disrupts these stages through persistent oxidative stress and inflammation [232,233]. Excessive ROS activates NF-κB, NLRP3, and TLR-MyD88 pathways, sustaining the release of IL-1β, IL-6, and TNF-α [234].

Increased M1 macrophages to pro-healing M2 macrophages polarisation further delays tissue repair [235]. AGE accumulation aggravates oxidative stress, endothelial dysfunction, and extracellular matrix degradation, impairing fibroblast activity, keratinocyte migration, collagen deposition, and angiogenesis [233]. Other diabetic skin complications include infections, acanthosis nigricans, diabetic dermopathy, blisters, and digital sclerosis [236]. Foot infections such as tinea pedis and onychomycosis may contribute to diabetic foot ulcers, which are further associated with neuropathy, poor circulation, impaired angiogenesis, chronic inflammation, barrier disruption, and polymicrobial infection [237,238,239].

Anthraquinone includes rhein and emodin. Rhein exhibits antioxidant and anti-inflammatory effects to relieve high-glucose-induced damage. When rhein is linked with chitosan, rhein amplifies its anti-inflammatory and antimicrobial properties, effectively decreasing tissue inflammation, improving keratinocyte growth as well as migration, and promoting diabetic wound healing [240,241]. Emodin relieves OS and inflammation induced by persistent hyperglycaemia [242]. It regulates the nuclear factor kappa B transcription factor (NF-κB) signalling pathway by suppressing the p65-NF-κB complex and increasing the conversion of pro-inflammatory M1 macrophages to pro-healing M2 macrophages. Furthermore, it enhances the levels of collagen III, fibronectin, and α-SMA, therefore accelerating wound healing through elevated ECM synthesis and granulation tissue formation [243].

TQ diminishes inflammation by inhibiting pro-inflammatory mediators and reducing oxidative stress at the wound site and helping in the formation of new tissue and the re-epithelialisation process. This is favourable for wound healing [244,245].

1,4-Naphthoquinone compounds and their derivatives, such as lawsone, juglone, shikonin, and plumbagin, have been discussed for wound healing potential due to their diverse structures, which contribute to preventing infections in wounds by their antibacterial properties. Various mechanisms of 1,4-naphthoquinone compounds have been reported in previous studies, including inhibition of protein and nucleic acid synthesis [246,247], apoptosis induction [248], alteration in redox processes [249], interference with cell wall synthesis, and inhibition of biofilm [250]. A prior clinical investigation has reported that rats with diabetes, when administered plumbagin, could develop enhanced wound healing activity. Between 10% and 20% plumbagin showed increased epithelialisation, collagen deposition, and increased state of antioxidant activity, and increased serum insulin levels. The inflammatory markers were reduced [245].

**Figure 5:**
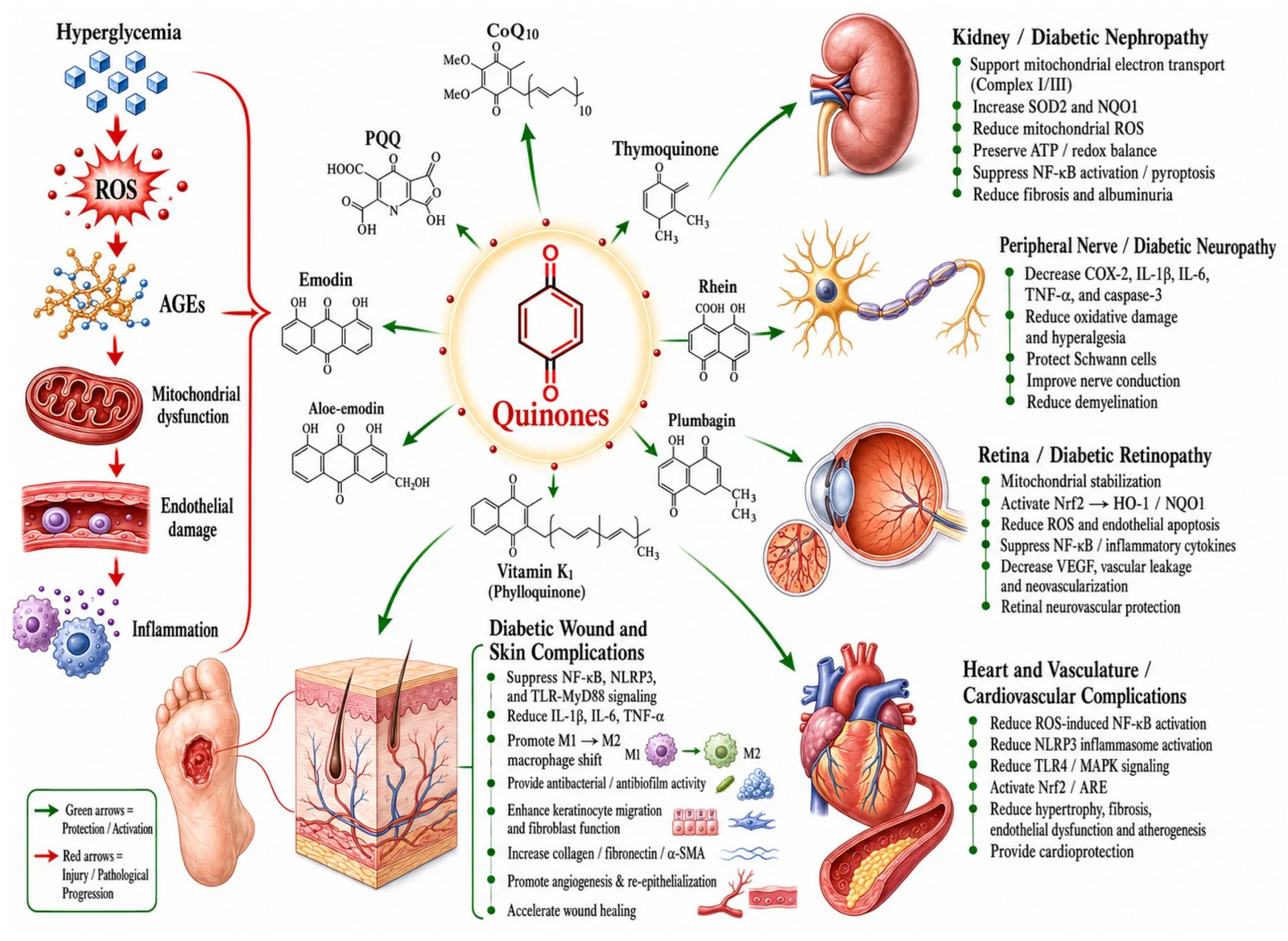
**Therapeutically relevant quinones and their principal biological actions.** This figure shows the overview of major quinone classes and representative compounds with therapeutic relevance. CoQ10/ubiquinone, endogenous quinones, vitamin K derivatives, thymoquinone, anthraquinones, juglone, plumbagin, lawsone, menadione, idebenone, atovaquone, and pixantrone/mitoxantrone. The central schematic highlights central mechanistic themes unifying quinone biology, i.e., modulation of reactive oxygen species, mitochondrial protection, and enhancement of antioxidant defences, suppression of inflammation, reduction of lipid peroxidation, improvement of mitochondrial integrity, and, in selected cases, inhibition of DNA synthesis. These combined features account for the increasing recognition of quinones as multifunctional scaffolds of relevance in metabolic, inflammatory, antimicrobial, cardiovascular, neuroprotective, wound-healing, and anticancer environments. The scientific information presented in the figure was synthesised from the published literature [177,178,179,180,181,182,183,184,185,186,187,188,189,190,191,192,193,194,195,196,197,198,199,200,201,202,203,204,205,206,207,208,209,210,211,212,213,214,215,216,217,218,219,220,221,222,223,224,225,226,227,228,229,230,231,232,233,234,235,236,237,238,239,240,241,242,243,244,245,246,247,248,249,250].

Mitochondrial dysfunction, excessive ROS formation, AGE–RAGE signalling, endothelial injury, and persistent inflammation appear repeatedly in the development of diabetes complications. Quinones may influence these processes, although the treatment requirements vary from one organ to another. Renal and cardiovascular applications require sustained systemic exposure, whereas retinal therapy depends on adequate ocular or retinal delivery. Neuropathy requires protection of neurons and Schwann cells, while diabetic wounds may benefit more from topical preparations that combine antimicrobial activity with inflammation control and tissue repair. For this reason, a positive finding in one complication should not be assumed to apply to another. Future studies should measure drug exposure within the target tissue and use outcomes that reflect the specific complication instead of relying solely on circulating antioxidant markers [242,243,244,245,246,247,248,249,250]. Quinones are actively involved in the management of diabetes and its potential complications through various mechanisms. To provide a broader pharmacological perspective, Figure 5 illustrates the main classes of therapeutically relevant quinones and how their shared redox-active framework leads to diverse biological and clinical applications, thus giving a broader pharmacological view. Redox and mitochondrial regulation unify the organ-protective potential of quinones, but the dominant pathological target and maturity of supporting evidence vary remarkably between individual diabetic complications (Supplementary Table S1).

## 6. Pharmacokinetics and Formulation Strategies

### 6.1. Absorption, Distribution, Metabolism, and Excretion (ADME)

Several factors affect ADME (absorption, distribution, metabolism, and excretion), the processes involved in pharmacokinetic studies [251]. Absorption is the process of transfer of a drug from the site of administration to the systemic circulation. Bioavailability is the fraction of the drug reaching the systemic circulation unchanged. Absorption can be affected by pH, lipid solubility, and membrane transporters [252]. Distribution describes drug movement from circulation into tissues and organs, while metabolism involves enzymatic conversion into metabolites. Excretion removes drugs from the body, primarily through urine and, to a lesser extent, bile, faeces, exhaled air, or sweat [253].

Although most of the quinones, such as CoQ10, VK1/2, and PQQs, demonstrate notable pharmacological properties, their clinical application is significantly hindered by their low bioavailability due to low aqueous solubility, rapid metabolism, and fast excretion [76]. CoQ10 is a benzoquinone hydrophobic compound with a large molecular weight, limiting its gastrointestinal absorption, leading to less than 5% oral bioavailability. In tissues, NAD(P)H-dependent quinone reductases convert CoQ10 into ubiquinol, which displays potent antioxidant, free radical-scavenging properties, and enhances mitochondrial efficiency in patients with T2DM. The metabolism of ubiquinol is predominantly influenced by both individual mitochondrial efficiency and age. Moreover, the solubility and absorption of CoQ10 are improved by emerging formulation strategies, including ubiquinol-based formulations, phytosomes, and nanoemulsions [76,254]. Idebenone is a synthetic analogue of CoQ10 with an enhanced pharmacokinetic profile. CoQ10 shows slower intestinal absorption, with a tmax of 6 to 8 h, while the synthetic analogue displays rapid absorption with a tmax of 1–2 h and rapid metabolisation [51].

Vitamin K is another type of significant quinone-based compound with a naphthoquinone parent structure that has received considerable research attention on its bioavailability. In the liver, it undergoes reduction and produces its active form, hydroquinone, which is crucial for γ-carboxylation reactions that participate in the metabolism of glucose and vascular health. The absorption of VK1 primarily occurs in the proximal part of the small intestine with the aid of bile acids, and its absorption is more efficient from vegetable oils compared to vegetables. However, it shows a low dietary absorption rate (5–15%) with a short half-life. VK1 can be recycled in the liver by redox cycling and can be recycled many times with a relatively low daily intake requirement. Compared to vitamin K1, vitamin K2 (menaquinones, MKs) has a longer iso-prenoid chain, resulting in better systemic retention and a lower clearance rate, thus leading to better bioavailability. Moreover, MK-7 demonstrates a prolonged half-life, which facilitates improved tissue distribution and sustained therapeutic benefits. The tissue distribution between VK1 and VK2 is notably different. VK1 is primarily found in the liver, pancreas, and heart, while the kidneys, brain, and lungs contain low levels. MK-4 is distributed throughout the body, but the liver, lungs, and heart contain low levels of MK-4. However, the bioavailability of vitamin K2 can be further improved by the growing new emulsification strategies [76,255,256].

PQQs (PQQ) exhibit very low oral bioavailability. After administration of PQQ, it reaches its peak serum level within 3 h. PQQ shows poor pharmacokinetic properties due to its hydrophilic characteristics and rapid systemic elimination, even though it displays potent redox cycling properties. Current formulation strategies, such as incorporating PQQ into liposomal carriers or food matrices, can enhance its absorption rate and circulatory stability. PQQ is metabolically stable, which means it undergoes minimal enzymatic metabolism and is primarily excreted through urine in its intact form [76,257].

Free anthraquinones have a higher ability to quickly absorb than conjugated glycosides due to their high lipid-soluble profile. Specifically, anthraquinones are primarily absorbed in the intestinal area rather than the stomach due to their high retention time in the intestines. The intestinal and hepatic circulation and reabsorption can lead to variations in blood concentration and multiple absorption peaks. However, anthraquinones display very low bioavailability and are extensively distributed throughout well-perfused tissues and organs such as the stomach, lung, intestine, heart, kidney, liver, and plasma. Mainly, it undergoes conjugation to glucuronides or sulfates, followed by excretion via urine or bile [76,258].

### 6.2. Bioavailability Barriers

The bioavailability of a drug plays a vital role in the therapeutic activity of the compound. It defines the rate and degree at which drug/active compounds are absorbed into the bloodstream after the drug administration through oral, topical, rectal, and parenteral routes. The bioavailability can be affected by several factors, such as the physicochemical properties, rate of absorption, metabolism, and excretion of the drug, and the route of administration. During drug development, the bioavailability of the drug should be considered in order to achieve effective therapeutic outcomes [259].

Quinones are compounds with a complex structure that may affect the bioavailability of the compounds. Therefore, the modification of the structure or addition of absorption promoters or alteration of drug dosage forms can enhance the bioavailability of the quinones. A previous study has shown that rhein has a carboxyl group in its structure, which can enhance its water solubility. Moreover, the appropriate lipid–water distribution coefficient can lead to the best bioavailability of the compound. However, the demethylation of physcion leads to poor bioavailability. Therefore, the structure and functional groups of the compound can also influence its bioavailability [260]. Moreover, anthraquinone has poor absorption, resulting in low bioavailability. Therefore, several nano-based drug delivery systems have been developed to enhance their bioavailability [261]. Moreover, the metabolism of drugs can also alter the bioavailability of the compounds. For instance, rhein, aloe-emodin, emodin, physcion, and chrysophanol are metabolised to sulfated conjugates, which can reduce the oral bioavailability of anthraquinones. Furthermore, quick demethylation can also be a factor for the poor bioavailability. Physcion shows poor bioavailability due to rapid demethylation into emodin. In addition, oxidation reactions can also reduce the bioavailability of anthraquinones [258].

Moreover, a drug’s structure can also affect its bioavailability. Among all the menaquinones, MK-7 is quickly and efficiently absorbed and thus leads to better bioavailability. MK-7 has a long chain and has a longer half-life in circulation, which results in longer exposure for absorption [262]. The bioavailability of crystalline CoQ10 is poor due to its large molecular weight and lipophilic nature. However, researchers are highly focused on new formulation strategies to improve their bioavailability. The oil-dispersed formulation, CoQ10-β-cyclodextrin powder, and nano-emulsion formulation of CoQ10 have shown better bioavailability than crystalline CoQ10 [263].

### 6.3. Toxicology

Although quinones have significant pharmacological properties, their notable toxicity, especially their carcinogenicity, hepatotoxicity, genotoxicity, and nephrotoxicity, remains a key issue. Quinones have the potential to damage the cellular membrane and DNA through the production of free radicals, which are toxic and covalently bind to cellular components, producing secondary free radicals and thereby inducing oxidative stress. Even though, at therapeutic doses, they display beneficial pharmacological effects with less toxicity, high doses or prolonged usage can trigger several adverse effects. For instance, some Chinese traditional medicines that possess anthraquinone have been associated with multiple adverse effects, including drug-induced kidney and liver injury. Moreover, quinones, including CoQ10, VK1, VK2, rhodopsin, and various plant-based compounds, are known as a double-edged sword due to their complex chemical structures that display both biological activity and potential toxicity. Recently, their toxicity has been widely studied due to their significant beneficial usage in nutritional and therapeutic formulations. Their therapeutic potential and cytotoxicity can vary depending on their unique chemical structures [76,264].

Quinone toxicity is not only harmful when it is exogenous but also when it is generated within our body during metabolism. Endogenous quinones can cause several diseases within our body. Our body can metabolise many dietary components, drugs, and inhalants and produce quinones, which are active metabolites and highly reactive, and can readily interact with biological macromolecules. For instance, troglitazone, a hypoglycaemic drug, is catalysed by CYP450 and peroxidase and produces troglitazone quinone, causing hepatotoxicity. Moreover, estrogen o-quinone is linked with breast cancer, and dopamine-o-quinones can cause Parkinson’s disease. In addition, quinones have been associated with other toxicities such as carcinogenesis, acute nephrotoxicity, and immunotoxicity [265,266].

Quinone derivatives act as strong toxins that cause genotoxic, cytotoxic, and immunotoxic outcomes. Phenol and catechol are metabolised by P450 peroxidase and produce quinone, which acts as a potent reactive electrophile. Lipids, proteins, and DNA can be damaged by quinones through the formation of ROS during redox cycling with relevant semiquinone radicals. Subsequently, this reaction paves the path to oncogenic mutation and the initiation of tumours [267].

A previous study conducted by D. Mtvralashvili et al. showed that 97 of 108 constipated patients with melanosis coli had a history of long-term consumption of anthraquinone laxatives [268]. The toxicity, such as genotoxicity and hepatotoxicity, caused by quinones may be due to oxidative stress. Especially, emodin-3-methyl ether increases ROS and leads to mitochondrial damage. Furthermore, quinone can also cause anuria, proteinuria, haematuria, and oliguria. Long-term or high-dose intake of quinone may lead to uremia, renal failure, and acute and chronic nephritis. For instance, high consumption of emodin disrupts glomerular filtration and tubular reabsorption, causing proteinuria. Furthermore, quinones are capable of crossing the placental barrier and subsequently cause toxic effects in the fetus [264]. They cause chromosomal aberrations or gene mutations by damaging the DNA in sperm cells, thus negatively affecting the quantity and quality of spermatozoa. A study conducted by K. Yao et al. concluded that prolonged exposure of male mice to N-(1,3-dimethylbutyl)-N′-phenyl-p-phenylenediamine-quinone can reduce their level of testosterone and semen quality, leading to impaired male fertility [269].

A pro-oxidant is any compound that can damage cells by promoting oxidative stress through the production of ROS or impairing antioxidant systems [270]. Under high concentrations and specific conditions, most antioxidants can act as pro-oxidants. Auto-oxidation of several antioxidants leads to the formation of free radicals, such as semiquinone and hydroxyl radicals, which are toxic and covalently bind to cellular components and produce secondary free radicals, thereby participating in pro-oxidant activity [271].

Moreover, quinones are potent redox compounds and display a pro-oxidant nature due to their participation in one-electron transfer reactions by P450/NADPH oxidoreductase 1 (NQO1), generating semiquinone radical anions, which are intermediate, unstable compounds that can subsequently react with oxygen to regenerate the original quinone and produce superoxide. This redox balance can impact antioxidant/pro-oxidant effects, thus affecting the cytoprotective/cytotoxic functional properties of quinones [272]. Plastoquinone, a natural quinone, is structurally similar to coenzyme Q10. The phosphonium derivative SkQ1 is a synthetic analogue of plastoquinone, which acts as a strong antioxidant at low concentrations while acting as a pro-oxidant at high concentrations (antioxidant/pro-oxidant ratio of 1000:1) [22]. Furthermore, idebenone also shows antioxidant and pro-oxidant properties through the formation of unstable semiquinone [273].

However, this redox cycling mechanism and pro-oxidant mechanism are highly employed in the treatment of cancer, causing oxidative stress through increasing ROS production and inhibiting the antioxidant system in cancer cells, thus killing the cancer cells. Conversely, the pro-oxidant mechanism can affect the normal cells, causing several toxicities and side effects. Therefore, a suitable drug delivery system should be developed to selectively target cancer cells [274].

### 6.4. Nanoformulations

Nanotechnology significantly revolutionised the pharmaceutical and medical fields at the end of the 20th century. Nanoformulation acts as either a carrier for the specific drug molecule to the targeted site or as a therapeutic agent. Many drugs have several limitations related to bioavailability, solubility, permeability, reaching the targeted site, toxicity, and other pharmacokinetic challenges, which can be overcome by the development of nanoformulations [275].

A previous study conducted by J. Taborsky et al. reported that TQ is one of the major significant bioactive compounds in *N. sativa* [276]. It has several pharmacological properties, including anticancer, antidiabetic, antiulcer, hepatoprotective, neuroprotective, and immunomodulatory effects. However, the progression of TQ into clinical trials has been hindered due to the challenges in formulation arising from its physicochemical properties and issues with oral administration. TQ is a lipophilic compound with low water solubility. Furthermore, it is unstable in aqueous medium and is strongly influenced by pH and light. The environment of the gastrointestinal tract and hepatic first-pass metabolism can influence the chemical and enzymatic alteration of the compounds. Consequently, the aqueous vehicle for TQ formulation and traditional oral drug administration failed to fulfil their therapeutic potential. Some techniques, such as the creation of nanoformulations, have been created to get over these hurdles [277,278].

Embelin, a benzoquinone compound isolated from *E. ribes*, shows several pharmacological properties, such as anticancer, antidiabetic, antioxidant, analgesic, and cardioprotective properties. However, based on the biopharmaceutics classification system (BCS), it is categorised as a class II drug due to its low solubility and high permeability [279]. A study conducted by Z. Li et al. reported that the oral bioavailability of embelin was low, at 30.2 ± 11.9% [280]. The reason for the low bioavailability is poor water solubility. Therefore, nanocarrier-based drug delivery has been significantly explored to overcome this challenge. Based on previous literature surveys, several nanorformulations, including liposomes, transfersomes, lipid nanospheres, chitosan NPs, gold NPs, and silver NPs, have been studied for embelin delivery in various disease conditions [279].

Lapachol, a naphthoquinone, displays anticancer, antibacterial, antiplasmodial, antifungal, anti-inflammatory, trypanocidal, and anti-angiogenic properties. Despite this, a previous clinical trial (phase I) showed poor therapeutic effect even at higher doses. To overcome this limitation, various drug delivery systems have been explored for better oral absorption and bioavailability. Nanoemulsions and microemulsions are the two main drug delivery systems, where both can be formulated as oil-in-water (O/W) or water-in-oil (W/O) based on the dispersed phase. Lapachol was effectively incorporated into O/W nanoemulsion, and an in vitro study proved that its sustained quinone release from nanoemulsion could be a promising therapeutic drug delivery system [281]. Previous studies reported that anthraquinones show anticancer properties by halting the growth of different cell lines and phases/cycle. For instance, 2-methyl-1,3,6-trihydroxy-9,10-anthraquinone, isolated from *Rubia yunnanensis*, demonstrated a potent anticancer property. However, anthraquinones present a barrier to the route of administration due to their low aqueous solubility and bioavailability. Based on previous research conducted on the encapsulation of red dye anthraquinone, it was demonstrated that nanocapsules increase the uptake of the drug by cells. In addition, the nanoformulation plays a major role in cancer treatment through improving the drug stability and high loading capacity for both hydrophobic and hydrophilic drugs, thus increasing the efficiency of drug delivery with a low dose of anticancer agents [282,283].

### 6.5. Advanced Drug Delivery Systems

Drug delivery systems are referred to as the formulation and storage of drug molecules into appropriate dosage forms, such as tablets or solutions, for administration, which can effectively reach the site of action and enhance therapeutic outcomes with fewer side effects. Drugs can be administered into the body through several routes based on their physiochemical properties and the targeted sites. Drug delivery systems should be considered for their improved drug solubility, drug efficacy, pharmacokinetic properties, and pharmacological properties, low toxicity, enhanced accumulation in target sites, and patient compliance [284].

Although quinone-derived compounds display promising therapeutic action, several barriers related to successful delivery remain challenging. Quinone displays poor aqueous solubility, poor absorption, fast metabolism, poor bioavailability, and toxicity, limiting its therapeutic effectiveness. Furthermore, the poor aqueous solubility of quinone significantly adversely affects its stability and bioavailability. Therefore, several strategies, such as structural modification, site-specific modification, and nanodelivery techniques, such as liposomes, micelles, biomimetic nanocarriers, and inorganic nanoparticles, have been explored to enhance the stability and solubility of quinone-derived drugs, leading to effective therapeutic outcomes. For instance, the bioavailability of chrysophanol can be improved by nanoparticles. The solubility of emodin hydrogel for topical application can be improved by the incorporation of Pluronic F-127 [76,285]. CoQ10 is commonly administered orally. However, its hydrophobicity and large molecular structure limit its retinal bioavailability. Vitamin E d-α-tocopheryl poly(ethylene glycol) 1000 succinate (TPGS), a water-soluble compound, is a vitamin E derivative. Conjugation of vitamin E TPGS and CoQ10 can increase the bioavailability of CoQ10 [286]. The therapeutic effectiveness of vitamin K can be enhanced by the formulation of nanocarriers for drug delivery [287].

### 6.6. Stability Enhancement Approaches

**Figure 6:**
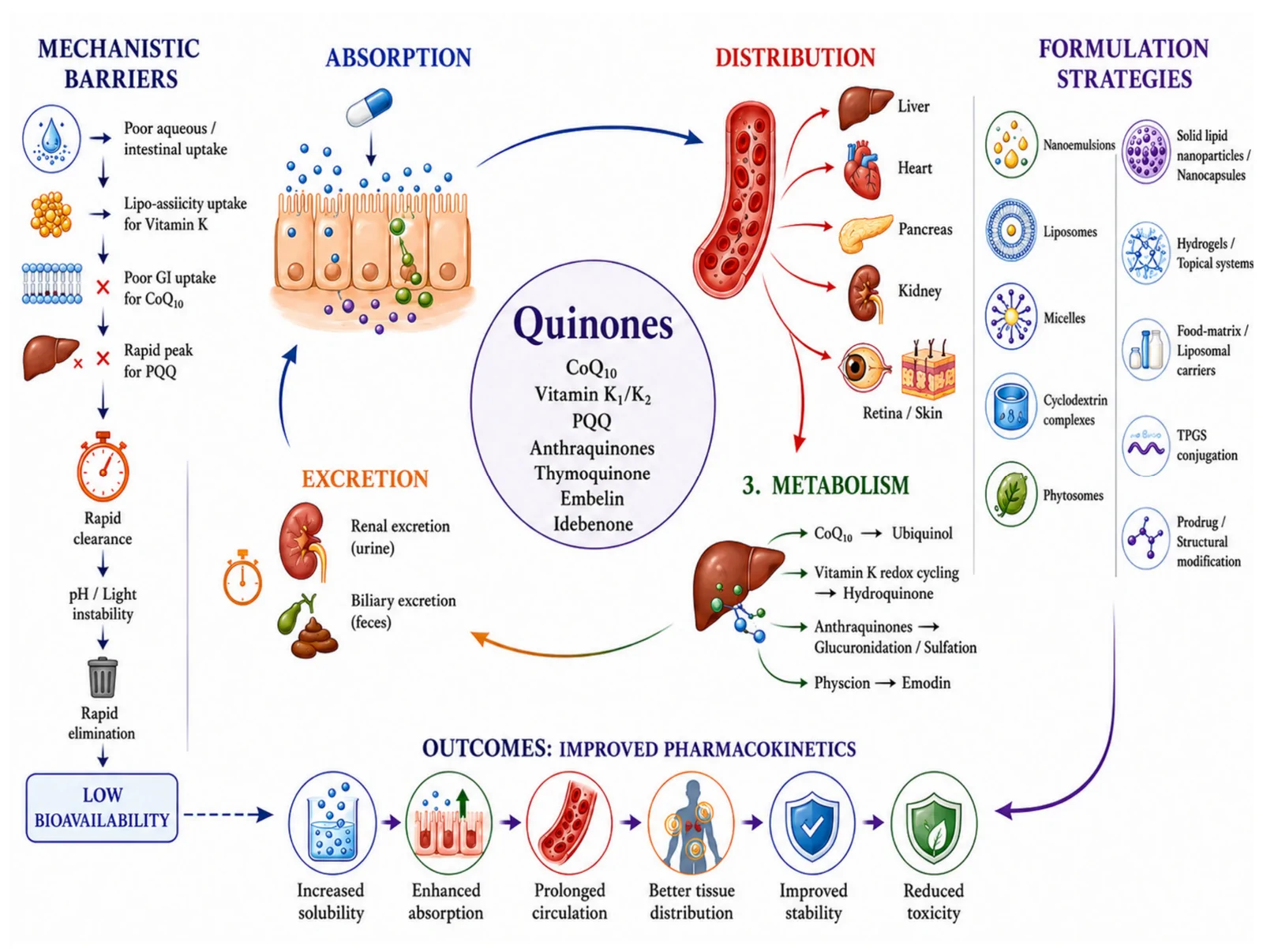
**Pharmacokinetic limitations and formulation strategies for improving quinone delivery.** This schematic summarises the absorption, distribution, metabolism, and excretion profiles of representative quinones, including CoQ10, vitamins K1/K2, pyrroloquinoline quinone, anthraquinones, thymoquinone, embelin, and idebenone. Their clinical translation is often restricted by poor aqueous solubility, limited gastrointestinal uptake, rapid clearance, metabolic transformation, and sensitivity to pH or light, resulting in low or variable bioavailability. Absorbed quinones distribute to metabolically active and disease-relevant tissues such as liver, heart, pancreas, kidney, retina, and skin prior to redox conversion glucuronidation, sulfation, or related metabolic processes and subsequent renal or biliary elimination. The scientific information presented in the figure was synthesised from the published literature [23,251,252,253,254,255,256,257,258,259,260,261,262,263,275,276,277,278,279,280,281,282,283,284,285,286,287,288,289,290,291].

Naturally occurring quinones are more stable. Generally, p-quinones display greater stability than o-quinones, as they possess a 1,2-diketone structure that reduces their stability. Many p-quinones, such as menadione and t-butylquinone, can be crystallised. Moreover, stable p-quinone can be found in several natural products, including juglone and ansamycin antibiotics, as well as in endogenous molecules, such as ubiquinone and vitamin K. Furthermore, many promising anticancer agents, such as diaziquone, daunorubicin, mitomycin C, doxorubicin, emodin, streptonigrin, and mitoxantrone, possess p-quinone in their structure, which provides the anticancerous property via the redox mechanism but leads to toxicity to normal cells due to ROS stimulation [23]. Even though aloins display several therapeutic activities, their rapid degradation in aqueous medium and poor solubility are considered major drawbacks for their clinical usage. Previous studies show that the storage stability of aloins and other anthraquinones can be enhanced through dehydration under different pH and temperature [288]. A study by D. Zhou et al. concluded that naphthoquinone pigment, which contains polyhydroxylated 1,4-naphthoquinone, are highly susceptible to degradation when exposed to high temperature and light. Therefore, it should be stored in a cool and dark area. This compound can be readily autoxidised when reacting with oxidising agents, alkaline conditions, and some metal ions, such as Fe^3+^, Al^3+^, Zn^2+^, and Ca^2+^ [289]. In addition, anthraquinones also display poor aqueous solubility and quick degradation during post-processing conditions, which affect their advantageous benefits. Emodin, aloe-emodin, rhein, chrysophanol, and physcion are five different anthraquinones that exhibit distinct thermal conditions. Among these quinones, emodin and aloe-emodin are highly sensitive to acidic and hydrolytic degradation, moderately susceptible to thermal (at 105 °C) and oxidative degradation, and less sensitive to basic degradation [290]. The encapsulation of vitamin K in micelles can enhance the stability of vitamin K in acidic conditions [291]. Formulation development should be guided by the main limitation of each quinone rather than by the general appeal of nanotechnology. CoQ10 and TQ are highly lipophilic and may benefit from lipid nanoparticles, nanoemulsions, phytosomes, or mixed micelles that improve dissolution and intestinal absorption. PQQ is hydrophilic but is removed rapidly from the circulatory system, so its formulation needs to improve cellular retention or prolong systemic exposure. Anthraquinones present a different problem because poor aqueous solubility is accompanied by chemical degradation and extensive glucuronidation or sulfation. For diabetic wounds, local delivery through hydrogels, nanofibres, or adhesive dressings may be more appropriate than systemic administration. Importantly, greater drug loading or apparent bioavailability does not by itself demonstrate better treatment. Formulation studies should also measure unbound drug concentrations, active metabolites, tissue distribution, release behaviour, and carrier-related toxicity [23,288,289,290,291]. Because the therapeutic performance of quinones is frequently limited by exposure rather than intrinsic activity, formulation selection should be matched to the dominant physicochemical barrier, intended route, and target tissue, as outlined in Supplementary Table S2. Figure 6 illustrates the pharmacokinetic journey of therapeutically relevant quinones and highlights how formulation-based interventions can overcome their major absorption, stability, distribution, metabolism, and elimination barriers. Because the therapeutic performance of quinones is frequently limited by exposure rather than intrinsic activity, formulation selection should be matched to the dominant physicochemical barrier, intended route, and target tissue.

## 7. Preclinical and Clinical Evidence

Increasing preclinical and emerging clinical evidence supports the therapeutic potential of quinones in diabetes management. Quinone-based compounds have been explored in different experimental platforms, from cell assays to animal disease models and humans, because of their redox-modulating, anti-inflammatory, and antimicrobial activities [292]. These studies suggest that quinones may have a role in the improvement of glycaemic control, reduction of oxidative stress, preservation of pancreatic β-cells, protection of vascular function, and wound healing [293]. However, the translation from bench to bedside is still challenging. Variability in compound structure, formulation, dosage, and study design have contributed to heterogeneous outcomes between studies. Several natural and synthetic quinones show promising bioactivity, but strong clinical validation remains limited [294]. Therefore, an assessment of the whole body of evidence from in vitro systems, animal models, and clinical trials is necessary to comprehend the therapeutic potential and current challenges of quinone-based interventions for diabetes management.

### 7.1. In Vitro Studies

Mechanistic understanding of the antidiabetic potential of quinones is based on in vitro studies. Quinones modulate multiple pathways involved in diabetic pathology in cellular studies using pancreatic β-cells, adipocytes, hepatocytes, endothelial cells, and immune cell models [295]. A consistently reported effect is the attenuation of oxidative stress by regulation of intracellular reactive oxygen species and enhancement of antioxidant defence systems such as superoxide dismutase, catalase, and glutathione-dependent enzymes [296].

Several quinones have been shown to have cytoprotective effects on pancreatic β-cells by reducing apoptosis and preserving the insulin-secretory function under hyperglycaemic stress. Quinones have been shown to enhance glucose uptake in insulin-resistant adipocyte and skeletal muscle models through the activation of AMP-activated protein kinase and insulin-sensitive signalling pathways, including PI3K/Akt-mediated GLUT4 translocation. Moreover, quinones inhibit inflammatory mediators like TNF-α, IL-6, and NF-κB, which help in decreasing chronic low-grade inflammation associated with metabolic dysfunction [297,298].

Quinones have demonstrated antimicrobial and antibiofilm activities against common wound pathogens and have been shown to promote fibroblast migration, collagen synthesis, and cellular proliferation in diabetic wound-related models [299]. Despite these encouraging results, in vitro systems are still reductionist models that cannot mimic the metabolic complexity, tissue interactions, and pharmacokinetic behaviour of living organisms. Thus, cellular findings should be taken as mechanistic evidence and not conclusive therapeutic validation.

### 7.2. Animal Models

Animal studies have substantially expanded the understanding of quinone-mediated therapeutic effects in diabetes and its complications. Experimental models such as streptozotocin-induced diabetes, alloxan-induced hyperglycaemia, db/db mice, and high-fat diet-induced insulin resistance have been widely employed to evaluate quinone efficacy in vivo [300,301]. Across these models, quinone-based interventions have demonstrated significant improvements in glycaemic control, insulin sensitivity, oxidative balance, and tissue protection.

Multiple studies report reductions in fasting blood glucose and glycated haemoglobin following quinone administration, suggesting improved metabolic regulation. Quinones have also been shown to preserve pancreatic islet architecture, reduce β-cell degeneration, and enhance endogenous insulin production [302]. Their antioxidant properties contribute to decreased lipid peroxidation and restoration of endogenous antioxidant enzymes in hepatic, renal, and vascular tissues [303].

In models of diabetic complications, quinones demonstrate protective effects against nephropathy, neuropathy, and delayed wound healing. Reduced inflammatory cytokine expression, improved angiogenesis, enhanced collagen deposition, and accelerated wound closure have been frequently observed [304]. Some quinone derivatives additionally display antimicrobial efficacy in infected diabetic wounds, supporting their dual therapeutic role [305]. Although animal models provide crucial translational evidence, species-specific metabolic differences and controlled experimental conditions limit direct extrapolation to human clinical outcomes.

### 7.3. Clinical Trials

Clinical evidence for quinone-based therapies in diabetes is still relatively limited but increasingly promising. Human studies have been predominantly performed on naturally derived quinones or quinone-containing formulations used as adjunctive therapies for glycaemic control, oxidative stress reduction, and management of diabetic complications. Preliminary clinical investigations indicate a beneficial effect on metabolic biomarkers, inflammatory parameters, and wound healing results [306].

A few quinone-rich compounds have been linked to modest decreases in fasting glucose, increased insulin sensitivity, and lowered markers of oxidative stress [307]. Quinone-related bioactive compounds in topical formulations have been shown to improve wound contraction, reduce microbial burden, and promote tissue regeneration in diabetic wound management [308]. These results indicate potential clinical utility, particularly in chronic wound care where multifunctional therapies are highly desirable.

However, the existing clinical literature is constrained by small sample sizes, short intervention durations, heterogeneous formulations, and limited standardisation of endpoints. In many studies, robust randomisation, placebo control, or multicentre validation was not employed. Moreover, the source and purity, as well as the delivery systems, of the quinone vary, making it difficult to compare results across studies. Thus, despite the promising clinical data obtained in the early studies, the support for quinones as therapeutic tools in the control of diabetes is still insufficient to allow their use as standard agents. Larger, well-designed randomised controlled trials are needed to determine long-term efficacy, optimal dosing, and safety.

### 7.4. Comparative Drug Efficacy

Current limitations of quinones for comparative evaluation against conventional anti-diabetic therapies.

Traditional anti-diabetic medicines, including metformin, sulfonylureas, insulin, and SGLT2 inhibitors, primarily target glycaemic control through specific metabolic pathways [309,310]. Quinones, however, provide a wider multimodal mechanism involving antioxidant, anti-inflammatory, antimicrobial, and cytoprotective actions besides metabolic modulation [311].

Such multifunctionality may offer a unique therapeutic advantage in complex diabetic complications, especially chronic wounds and inflammatory tissue damage, where hyperglycaemia is only one component of disease progression. Quinones can be used in combination with existing drugs by focusing on oxidative and inflammatory pathways not directly targeted by conventional glucose-lowering agents [23]. Some preclinical studies have demonstrated synergistic effects of quinones with standard therapies, improving overall therapeutic response and possibly reducing drug doses [312].

Nevertheless, there are few direct head-to-head comparative studies. At present, the data do not yet support the replacement of conventional therapies by quinones as monotherapy. Instead, quinones seem at present more suitable as adjunct or combinatorial therapeutic agents [313]. Comparative pharmacokinetic, efficacy and safety studies are needed to determine their relative clinical value and to define therapeutic positioning in diabetes treatment algorithms.

### 7.5. Translational Limitations

Despite considerable preclinical success, several barriers impede the clinical translation of quinone-based therapeutics for diabetes. A major challenge is the dual redox nature of quinones. Although regulated redox activity can have therapeutic benefits, excessive redox cycling can produce cytotoxic reactive oxygen species, leading to off-target oxidative injury [314]. This narrow therapeutic window makes dose optimisation and safety evaluations more difficult. Pharmacokinetic limitations further limit clinical utility. Many quinones have poor aqueous solubility, limited oral bioavailability, rapid metabolism, and unfavourable tissue distribution [315]. These properties may lead to decreased therapeutic efficiency and require advanced formulation strategies for efficient delivery. The variability in biological activity is also due to structural diversity among quinones, which makes it difficult to generalise across compounds [316].

A further critical limitation is the absence of standardised experimental frameworks. Differences between model systems, dosage regimens, formulations, and outcome measures make reproducibility difficult and evidence synthesis complicated. Regulatory challenges persist, especially for natural product-derived quinones where batch consistency and quality control are still problematic. Finally, long-term human safety data are still lacking. Long-term use may carry risks of redox imbalance, hepatotoxicity, or other undesirable metabolic effects [317]. To fully realise the therapeutic potential of quinones in diabetes care, integrated efforts involving medicinal chemistry, pharmaceutical engineering, mechanistic toxicology, and rigorous clinical trial design will be required to overcome these translational barriers.

When the evidence is stratified according to study design and direct relevance to diabetes, clinical support is strongest for CoQ10, whereas most other quinones remain at mechanistic, preclinical, or indirect human-evidence stages (Table 3). A clear gap remains between the extensive mechanistic evidence for quinones and the amount of reliable clinical evidence available. CoQ10 has been examined in systematic reviews, meta-analyses, and human supplementation studies, with modest improvements reported in some measures of glycaemic control, inflammation, and oxidative stress [316]. Observational research has linked vitamin K intake with diabetes risk, but the association does not prove that vitamin K supplementation can prevent or treat the disease [317]. PQQ has produced measurable effects on inflammation and mitochondrial metabolism in human participants, although it has not been adequately tested as a treatment for diabetes [318]. Evidence for TQ, anthraquinones, naphthoquinones, and synthetic mitochondria-targeted compounds still come largely from cell and animal experiments. Therefore, these agents should be referred to as experimental or potential adjunctive therapies and not as clinical antidiabetic agents.

## 8. Future Perspectives, Limitations, and Research Directions

Quinones remain of interest as potential modulators of oxidative stress, insulin signalling, and diabetes-associated tissue injury. However, individual compounds show substantial variation in the available evidence, and most quinones, except for CoQ10, have not been adequately evaluated in well-controlled human studies. Future research should therefore focus on defining pharmacologically relevant concentrations, improving bioavailability and tissue targeting, establishing long-term safety, and determining whether experimental findings can be reproduced in clinically relevant settings [23,311,312]. To facilitate full-scale developments in understanding how quinones can protect β-cells, improve insulin sensitivity, and ameliorate limitations associated with diabetic nephropathy, neuropathy, and retinopathy in diabetic patients, there is a clear need for comprehensive research that integrates redox biology, medicinal chemistry, and animal models of specific diseases; therefore, drug delivery systems, the identification of at-risk patients, and biomarkers of oxidative stress and improved metabolism will be crucial for advancing to phase-one clinical trials [312,313,314,315,316]. Finally, it is clear that to ensure the maximisation of medicinal developments by quinones and their full potential as new treatments for the long-term management of diabetes and its related complications, a specific level of collaborative research effort across various disciplines is essential [317,318].

### 8.1. Targeted Drug Design and Quinone Derivatisation

Quinones provide a versatile chemical framework for exploring new approaches to metabolic disease, largely because their redox properties can influence several cellular pathways involved in diabetes. However, the same redox activity that contributes to their biological effects may also produce unwanted oxidative or electrophilic stress, depending on the structure and concentration of the compound. This redox activity makes structural optimisation an important part of quinone drug development [319]. On the exact opposite side is their potential as antioxidants, which can contribute significantly to reducing the effects of oxidative stress and play a significant role in contributing to the cause and development of metabolic disorders. It is important to note that quinones have exemplary potential in energy production and catalysis by enzymes, which can be beneficial for treating diseases like diabetes mellitus, as these conditions significantly affect metabolic pathways. At present, research work is focused on finding the means of utilising the exemplary potential of quinones. With that aim in mind, there arises a focus on understanding their significance in metabolic pathway mechanisms.

Designing and derivatising quinones appears to be important for developing superior non-toxic drugs. In this context, the current scientific approach aims at the rational designing of the structure of quinones for enhancing their specificity against diabetes, non-specific electrophilic binding, and redox potential. The derivatisation of the structure of the quinone molecules through the addition of electron-withdrawing or electron-donating heterocyclic substitution and/or prodrug linkages enables the enhancement of the ability of the quinone molecules to optimise stability, solubility, and targeting of mitochondria, respectively, without compromising the antioxidant properties and mechanisms of quinones against DM [320]. The scientific computational models for docking identify the highly interacting derivatives of the quinone molecules for determining their binding properties with the target biological pathways of Nrf2/Keap1, AMPK, aldose reductase, and α-glucosidase pathways, respectively. On the basis of the published scientific literature, the binding of the quinone molecules to the molecules of peptides and sugars, and the carrier molecules of nanoparticles, enables the enhancement of targeting of the tissue, including the liver, pancreas, kidney, and retina, and inhibits systemic oxidative stress. Scientifically, both individually and in combination, all the designs broaden the possibilities for developing next-generation derivatives of quinone molecules that aim to achieve superior potential by reducing the toxicity and adverse effects associated with their administration in the treatment of DM complications [321].

### 8.2. Integration with Nutraceutical and Functional Food Approaches

Recent studies suggest that the use of quinones in functional foods and nutraceuticals could represent a safe and effective method for harnessing their potential to treat DM. There is evidence that compounds such as quinones, which are derived from plant sources, or coenzyme Q10, may provide a basis for this justification, as they can potentially alter redox regulation in cells, as well as the regulation of insulin and mitochondrial functions. However, their application in functional foods and nutraceuticals may provide a valid justification for their safe use in the body, rather than their use as drugs that react to specific needs [322]. Among the considered food delivery systems are food emulsions comprising liposomes and polysaccharide gels, which may increase the bioavailability of quinones that may become effective when administered as foods rather than as drugs; reacting to their needs inside the body may increase their effective use, as already discussed in a recent study that took into consideration using food emulsions that can increase their bioavailability of up to 30% [323]. Furthermore, using quinones may show positive use when applied together with other substances, such as antioxidants derived from polyphenol vitamins or trace elements, that can show effective use regarding their positive side when applied together, as already discussed in a recent study that considered the positive use of such substances [324].

### 8.3. Multi-Targeted Therapeutic Potential of Quinones

Quinones are a diverse class of bioactive molecules that demonstrate various pharmacological activities and can facilitate redox cycling. Based on their unique chemical structure, they can bind to multiple targets. These molecules can exhibit antioxidant, pro-oxidant, anti-inflammatory, anticancer, antimicrobial, and cardioprotective actions, depending on the biological environment [325]. Their ability to alter redox cycling in mammalian cells, regulate mitochondrial activity, induce apoptosis in cancer cells, and modulate multiple signalling pathways has drawn considerable attention in the scientific community. Research on the discovery of drugs targeting non-communicable diseases, infectious diseases, metabolic disorders, and neurological disorders is exploring the role of natural quinones, including ubiquinones, benzoquinones, and naphthoquinones, as well as synthetic quinones. The pathways that these molecules operate upon include scavenging of ROS, induction of antioxidant response elements (for example, Nrf2), suppression of pro-inflammatory pathways (NF-κB), modulation of AMPK, or actions on the insulin signalling pathways [326].

### 8.4. Omics-Based and Systems Biology Approaches to Elucidate Mechanisms

DM is a multifactorial disorder influenced by genetic and environmental factors that disrupt glucose metabolism, cellular redox balance, and inflammatory pathways. Quinones, including benzoquinones, naphthoquinones, and anthraquinones, have attracted interest because of their structural diversity and potential to modulate oxidative stress, mitochondrial function, aldose reductase, insulin signalling, inflammatory mediators, and glucose and lipid metabolism [327]. Omics approaches, including transcriptomics, proteomics, and metabolomics, can further clarify how quinones influence diabetes-related molecular and metabolic pathways and help identify system-level responses and therapeutic targets [328,329]. However, translation calls for careful assessment of bioavailability, pharmacokinetics, safety, and reproducibility. Thus, development should start with defining therapeutic concentrations that provide redox protection but do not cause oxidative or electrophilic injury, followed by assessment of systemic and tissue exposure, mitochondrial function, Nrf2 activation, carbonyl stress, and insulin signalling. The delivery systems should be designed depending on the target organ and the physicochemical properties. Natural quinones also need to be routinely checked for identity, purity, stability, and batch-to-batch consistency. Then, promising candidates need to be tested in controlled clinical studies with standardised formulations, adequate comparators, and clinically relevant outcomes. The therapeutic potential of quinones in diabetes will depend heavily on interdisciplinary cooperation [326,327,328,329].

## 9. Current Updates on Different Types of Studies on Quinones as Antioxidant and Antidiabetic Agents in Diabetes

Recent research has examined quinones as potential modulators of oxidative stress, glucose metabolism, and diabetes-associated tissue injury, but the strength of evidence differs considerably among individual compounds and study types [330,331,332,333,334].

CoQ10 has been evaluated more extensively in human studies than most other quinones, including systematic reviews, meta-analyses, and supplementation trials. These studies have reported modest improvements in selected measures of glycaemic control and oxidative stress, although the findings are not uniform across all studies and formulations [335,336,337,338,339]. Evidence for other quinones, including TQ, remains more dependent on experimental models, where effects on oxidative stress, inflammation, insulin signalling, and tissue protection have been reported.

Several statistical and population-based studies have examined the relationship between oxidative stress, glycaemic control, and the development of diabetic complications. These studies generally support an association between greater oxidative burden, impaired metabolic control, and complications such as nephropathy and neuropathy [340,341,342,343,344]. Such findings are useful for understanding the biological context in which quinones may act, but they do not by themselves establish that quinone supplementation can prevent or treat diabetes-related complications. Similarly, observational studies involving dietary or circulating quinone-related compounds may identify associations with metabolic outcomes, but these findings should not be interpreted as evidence of therapeutic efficacy.

Clinical intervention studies provide more direct evidence, although their findings need to be considered according to the specific quinone, study population, dose, formulation, and measured outcome. CoQ10 has been investigated in human supplementation studies, with some reports describing improvements in glycaemic measures, inflammatory markers, and oxidative stress [335,336,337,338,339]. Other clinical investigations have examined outcomes such as endothelial function, blood pressure, and wound-related parameters [345,346,347,348]. These findings indicate that selected quinones may have biological effects relevant to diabetes and its complications, but they do not establish a class-wide therapeutic effect. In particular, one should not interpret improvements in biomarkers or secondary clinical outcomes as evidence that quinones can replace established glucose-lowering therapies.

The evidence for TQ, anthraquinones, naphthoquinones, and mitochondria-targeted quinone derivatives remains less mature. Much of the available evidence for these compounds comes from cellular and animal models, where antioxidant, anti-inflammatory, insulin-sensitising, and organ-protective effects have been described [299,300,301,302,303,304,305,306,307]. PQQ has also shown measurable effects on inflammatory and mitochondrial-related pathways in human participants, but diabetes-specific therapeutic efficacy has not yet been adequately established [296,297,298,299]. Similarly, observational evidence concerning vitamin K and diabetes risk should be interpreted as an association, rather than proof that supplementation can prevent or treat diabetes [295]. These distinctions are important when considering the translational relevance of experimental findings.

Overall, the current literature provides a rationale for continued investigation of quinones in diabetes, particularly as potential adjunctive or experimental approaches (Table 4). However, the available evidence does not support treating quinones as a replacement for conventional antidiabetic therapies. The main limitations include small study populations, short intervention periods, differences in quinone preparations and doses, heterogeneous formulations, and limited long-term safety and efficacy data. The absence of sufficient head-to-head clinical studies also prevents firm conclusions about the comparative effectiveness of quinones relative to established treatments. Larger, well-designed, multicentre, randomised, controlled trials using standardised preparations, clearly defined doses, appropriate comparators, and clinically meaningful outcomes are needed to determine which quinones, if any, can provide reproducible benefits in patients with diabetes [23,349,350,351,352].

**Table 4:** Recent evidence from systematic reviews, meta-analyses, statistical analyses, cohort studies, and randomised controlled trials evaluating quinones as antioxidant and antidiabetic agents in diabetes.

Type of Study	Objective	Key Outcomes	References
**Systematic review**	Evaluate antioxidant and glycaemic effects of CoQ10 in diabetic patients	CoQ10 improved oxidative stress biomarkers and modestly reduced fasting glucose and HbA1c	[330]
	Review TQ-mediated metabolic protection	Demonstrated antioxidant, anti-inflammatory, and insulin-sensitising properties	[331]
	Assess quinone phytochemicals in diabetic complications	Highlighted ROS suppression, AMPK activation, and β-cell protection	[332]
	Summarise quinone-based antioxidant therapeutics	Reported reduced lipid peroxidation and inflammatory signalling	[333]
	Examine therapeutic potential of natural quinones	Strong evidence for antioxidative and mitochondrial protection	[334]
**Meta-analysis**	Quantify effects of CoQ10 on glucose metabolism	Significant reductions in fasting glucose and HbA1c	[335]
	Evaluate antioxidant biomarkers	Increased SOD and TAC; decreased MDA	[336]
	Compare antioxidant interventions	Quinone-based supplements improved oxidative balance	[337]
	Analyse insulin sensitivity outcomes	Reduced HOMA-IR significantly	[338]
	Aggregate intervention studies	Confirmed consistent ROS-lowering effects	[339]
**Statistical analysis**	Correlate antioxidant biomarkers with diabetes prevalence	Lower circulating CoQ10 associated with higher oxidative burden in T2DM	[340]
	Study redox biomarkers and diabetes risk	Reduced antioxidant reserve predicted diabetes progression	[341]
	Statistical association of oxidative biomarkers	ROS positively correlated with HbA1c and insulin resistance	[342]
	Quantify oxidative stress burden	Quinone-linked antioxidant pathways reduced inflammation	[343]
	Examine oxidative markers in diabetic complications	High oxidative stress associated with nephropathy and neuropathy	[344]
**Cohort study**	Assess long-term antioxidant supplementation	Improved glycaemic stability and oxidative status	[345]
	Monitor quinone-rich diet and diabetes incidence	Higher quinone intake linked to lower T2DM risk	[346]
	Evaluate dietary quinones	Reduced inflammation and improved insulin sensitivity	[346]
	Study antioxidant micronutrients and metabolic disease	Antioxidant-rich diet associated with reduced diabetes progression	[347]
	Examine oxidative biomarkers longitudinally	Persistent oxidative stress predicted complications	[348]
**Randomised controlled trial**	Evaluate glycaemic effects of CoQ10	Improved HbA1c and blood pressure	[349]
	Assess vascular protection	Improved endothelial function and reduced oxidative damage	[350]
	Study renal and metabolic effects	Improved insulin metabolism and inflammatory markers	[351]
	Evaluate antioxidant efficacy	Reduced MDA and inflammatory cytokines	[352]
	Assess wound healing efficacy	Accelerated wound closure and reduced infection burden	[23]

## 10. Conclusions

Quinones impact several processes relevant to diabetes, including mitochondrial energy production, oxidative stress, insulin resistance, β-cell injury, glycation, and inflammation. These properties provide a justification for their study in both glycaemic control and diabetic complications. At the same time, their redox activity is an important challenge. At one concentration, a quinone may be protective; at another, it may produce ROS or bind to cellular molecules non-specifically. Other barriers are poor solubility, rapid metabolism, and uneven tissue exposure. While the clinical benefits appear to be moderate and dependent upon formulation and dose, CoQ10 has the most extensive human data of all the chemicals assessed. Most other quinones are supported largely by laboratory and animal studies. They should therefore be considered as possible adjunctive agents rather than substitutes for established diabetes therapies. Future progress will be determined by safer structural derivatives, formulations for specific organs, reliable pharmacokinetic and redox biomarkers, and well-controlled clinical trials. The resolution of these issues will determine if quinone chemistry can be translated into viable therapies for diabetes-associated metabolic and tissue damage.

## Figures and Tables

**Table 2:** Mechanism-to-outcome map of quinone-mediated metabolic regulation in diabetes.

Pathological Node	Representative Quinones	Principal Molecular Actions	Expected Metabolic Outcomes	Current Evidence Maturity	References
β-Cell oxidative and mitochondrial injury	CoQ10, PQQ, MitoQ, thymoquinone	Preservation of mitochondrial function; reduction of lipid peroxidation and ROS; activation of Nrf2; suppression of NF-κB and apoptosis	Preserved β-cell mass; improved glucose-stimulated insulin secretion; and reduced glucotoxicity	Predominantly cellular and animal evidence; limited diabetes-specific human validation	[104,105,110,113,114,115]
Insulin resistance and impaired glucose uptake	β-Lapachone, tanshinone IIA, plumbagin, sennidin A, rhinacanthin C	Activation of NQO1–AMPK, PI3K/Akt, and AKT/mTOR; increased GLUT4/GLUT2 translocation	Increased glucose uptake in skeletal muscle and adipose tissue; improved insulin sensitivity	Mainly in vitro and animal evidence	[118,119,120,121,122,123,124,125]
Defective insulin-receptor signalling	Demethylasterriquinone B1, emodin, thymoquinone, embelin	Insulin-receptor activation; IRS-1/PI3K/Akt phosphorylation; AMPK–SIRT1-associated regulation	Restoration of insulin responsiveness and suppression of hepatic gluconeogenic signalling	Strong mechanistic evidence but limited comparative clinical evidence	[129,130,131,132,133,134,135,136,137,138,139,140,141,142]
Postprandial hyperglycaemia	Embelin, emodin, aloe-emodin, alaternin, naphthoquinone derivatives	Inhibition of α-amylase and α-glucosidase; delayed carbohydrate hydrolysis	Reduced intestinal glucose release and attenuation of postprandial glycaemic excursions	Primarily biochemical and in vitro evidence	[145,146,147,148,149]
AGE–RAGE and carbonyl stress	Emodin, rhein, aloe-emodin, thymoquinone, PQQ, lawsone	Carbonyl trapping; metal chelation; radical scavenging; inhibition of AGE–RAGE signalling	Reduced glycoxidative injury; inflammation and extracellular-matrix cross-linking	In vitro and preclinical evidence; limited human mechanistic data	[158,159,160,161,162,163]
Gut dysbiosis and metabolic endotoxaemia	Vitamin K/menaquinones, emodin, rhein, aloe-emodin	Microbial electron transfer; ecological cross-feeding; possible modulation of AGE–RAGE-related microbiota changes	Potential improvement in barrier function, SCFA signalling, and insulin sensitivity	Preliminary and largely indirect; causal evidence remains insufficient	[103,175,176]

**Table 3:** Evidence maturity, clinical directness, and claim boundaries for quinone-based interventions in diabetes.

Intervention/Evidence Group	Evidence Represented in the Manuscript	Directness to Diabetes	Scientifically Supportable Conclusion	Claim That Should Be Avoided	References
CoQ10 supplementation	Systematic reviews and meta-analyses of glycaemic outcomes	Direct, although study populations and formulations vary	CoQ10 may produce modest improvements in selected glycaemic and oxidative stress outcomes	CoQ10 is an established glucose-lowering treatment	[292]
CoQ10 and inflammatory/oxidative biomarkers	Umbrella-level synthesis and broader clinical studies	Partly indirect	Supplementation may improve selected inflammatory and oxidative biomarkers	Biomarker improvement necessarily prevents diabetic complications	[293]
CoQ10 in T2DM complications	Observational association with oxidative stress, neuropathy, and cardiovascular disease	Direct but non-causal	CoQ10 status is associated with oxidative burden and complications in T2DM	Low CoQ10 is proven to cause neuropathy or cardiovascular disease	[294]
Dietary vitamin K exposure	Prospective cohort analysis of vitamin K intake and incident T2DM	Direct observational evidence	Higher dietary vitamin K exposure may be associated with lower T2DM risk	Vitamin K supplementation prevents or treats diabetes	[295]
PQQ supplementation	Human mechanistic study of inflammation and mitochondrial-related metabolism	Indirect; not diabetes-specific	PQQ has human biological activity relevant to redox and mitochondrial regulation	Clinical antidiabetic efficacy has been demonstrated	[296,297,298,299]
TQ and plant anthraquinones	Narrative reviews, cellular experiments, and animal diabetes models	These compounds show antioxidant, anti-inflammatory, insulin-sensitising, and organ protective effects in experimental models	These compounds show plausible insulin sensitising, antioxidant, and organ-protective effects	Clinical efficacy and long-term safety in diabetes remain insufficiently established	[299,300,301,302,303,304,305,306,307]
Vitamin K and wound healing	Systematic review and meta-analysis of preclinical studies	Experimental studies support possible roles in wound-repair mechanisms	Vitamin K supports experimental wound repair mechanisms	Clinical efficacy in diabetic wound healing requires further direct investigation	[23,308,309,310,311]

## Data Availability

No new data were created or analyzed in this study. Data sharing is not applicable to this article.

## Supplementary Materials

The following supporting information can be downloaded at: https://www.mdpi.com/article/10.3390/pharmaceutics18091191/s1, Table S1: Organ-specific therapeutic landscape of quinones in diabetic complications: pathological targets, candidate compounds and translational gaps. Table S2: Barrier-driven formulation strategies for translating quinone pharmacology into diabetes-directed therapy.

## Abbreviations

The following abbreviations are used in this manuscript:

ADME	Absorption, distribution, metabolism, and excretion
AGE	Advanced glycation end-product
AICAR	5-aminoimidazole-4-carboxamide ribonucleoside
AMPK	AMP-activated protein kinase
ANQs	Anthraquinones
AKt	Protein kinase B
AP1	Activator protein 1
ARE	Antioxidant response element
ATP	Adenosine triphosphate
CaMKK	Calcium/calmodulin-dependent protein kinase kinase
CoQ10	Coenzyme Q10
DNA	Deoxyribonucleic acid
DPPH	2,2-diphenyl-1-picrylhydrazyl
DM	Diabetes mellitus
ECM	Extracellular matrix
ELISA	Enzyme-linked immunosorbent assay
ER	Endoplasmic reticulum
ERK	Extracellular signal-regulated kinase
FBG	Fasting blood glucose
FOXO	Forkhead box O
FRAP	Ferric reducing antioxidant power
GLUT2 GLUT4	Glucose transporter type 2 Glucose transporter type 4
GPx	Glutathione peroxidase
HFD/STZ	High-fat diet/streptozotocin
HDL-C	High-density lipoprotein cholesterol
HepG2	Human hepatocellular carcinoma cell line
IL-2	Interleukin-2
JAK/STAT	Janus kinase/signal transducer and activator of transcription
LC-MS	Liquid chromatography–mass spectrometry
LDL-C	Low-density lipoprotein cholesterol
MAPK	Mitogen-activated protein kinase
MitoQ	Mitoquinone
MTAQ	2-methyl-1,3,6-trihydroxy-9,10-anthraquinone
NF-κB	Nuclear factor kappa B
NQO1	NAD(P)H:quinone oxidoreductase 1
Nrf2	Nuclear factor erythroid 2–related factor 2
NAFLD	Non-alcoholic fatty liver disease
NAD(P)H	Nicotinamide adenine dinucleotide phosphate
NLRP3	NOD-like receptor family pyrin domain-containing 3
PI3K	Phosphoinositide 3-kinase
PQQ	Pyrroloquinoline quinone
PPAR	Peroxisome proliferator-activated receptor
PTP1B	Protein tyrosine phosphatase 1B
RAGE	Receptor for advanced glycation end-products
ROS	Reactive oxygen species
SCFAs	Short-chain fatty acids
HOMA-IR	Homeostatic model assessment of insulin resistance
HSA	Human serum albumin
Sirt3	Sirtuin 3
SIRT1	Sirtuin 1
TPP	Triphenylphosphonium
VEGFA	Vascular endothelial growth factor A
TBARS	Thiobarbituric acid-reactive substances
T2DM	Type 2 diabetes mellitus
