# Supplementary material for: Translational Pharmaceutical Perspectives on Quinones as Antioxidant and Antidiabetic Agents in Diabetes and Its Complications
Source: Pharmaceutics. 2026 Sep 20;18(9):1191. doi: 10.3390/pharmaceutics18091191 (PMC13609996; doi:10.3390/pharmaceutics18091191)
Supplement: Supplementary file 1 [file pharmaceutics-18-01191-s001.zip › pharmaceutics-4471528-supplementary.pdf]

Supplementary Table S1. Organ-specific therapeutic landscape of quinones in diabetic complications: pathological targets, candidate compounds and translational gaps.

Diabetic complication	Dominant pathological processes	Candidate quinones	Proposed protective mechanisms	Relevant outcomes	Major translational gap	References
Diabetic nephropathy	Mitochondrial dysfunction, ROS accumulation, NF-κB activation, pyroptosis and fibrosis	CoQ10, PQQ, TQ	Restoration of electron transport, suppression of mitochondrial ROS and inhibition of NF-κB/pyroptosis signalling	Albuminuria, renal function, fibrosis, mitochondrial membrane potential and oxidative biomarkers	Lack of standardized renal exposure data and adequately powered renal-outcome trials	[1, 2]
Diabetic neuropathy	Oxidative injury, Schwann-cell apoptosis, demyelination and neuroinflammation	TQ, PQQ, CoQ10, vitamin K1	Reduction of COX-2, IL-1β, IL-6, TNF-α and lipid peroxidation; preservation of Schwann cells and nerve conduction	Neuropathic pain, nerve-conduction velocity, demyelination and inflammatory markers	Limited human evidence beyond adjunctive supplementation	[3, 4]
Diabetic retinopathy	Endothelial injury, mitochondrial ROS, vascular leakage, VEGF signalling and retinal neurodegeneration	CoQ10, short-chain mitoprotective quinones, aloe-emodin	Mitochondrial stabilization, Nrf2/NQO1/HO-1 activation, NF-κB inhibition and VEGF suppression	Retinal leakage, MMP-9, retinal thickness, ganglion-cell survival and visual function	Ocular pharmacokinetics, retinal exposure and long-term safety remain poorly defined	[5, 6]
Diabetic cardiovascular injury	Endothelial dysfunction, NLRP3 activation, myocardial fibrosis and impaired mitochondrial bioenergetics	CoQ10, PQQ, tanshinones, embelin, plumbagin, aloe-emodin	Nrf2 activation; NF-κB/NLRP3 inhibition; mitochondrial preservation and improved NO signalling	Endothelial function, myocardial fibrosis, cardiac function, oxidative stress and inflammation	Few diabetes-specific cardiovascular endpoint trials	[7, 8]
Diabetic wounds and skin injury	Persistent M1 macrophage activation, infection, impaired angiogenesis, ECM loss and oxidative stress	Rhein, emodin, TQ, plumbagin, lawsone, juglone, shikonin	M1-to-M2 macrophage transition, antimicrobial and antibiofilm activity, collagen deposition, keratinocyte migration and re-epithelialization	Wound-closure rate, microbial burden, collagen organization, angiogenesis and inflammatory markers	Formulation heterogeneity and scarcity of randomized human wound trials	[9, 10]

References

1. Jha R, Lopez-Trevino S, Kankanamalage HR, Jha JC. Diabetes and Renal Complications: An Overview on Pathophysiology, Biomarkers and Therapeutic Interventions. *Biomedicines.* **2024**;*12*(5). doi:10.3390/biomedicines12051098
2. Tirichen H, Yaigoub H, Xu W, Wu C, Li R, Li Y. Mitochondrial Reactive Oxygen Species and Their Contribution in Chronic Kidney Disease Progression Through Oxidative Stress. *Front Physiol*. **2021**;*12*:627837. doi:10.3389/fphys.2021.627837 PubMed PMID: 33967820.
3. Zhang Z, Huang Q, Zhao D, Lian F, Li X, Qi W. The impact of oxidative stress-induced mitochondrial dysfunction on diabetic microvascular complications. *Front Endocrinol.* **2023**;14(February):1–15. doi:10.3389/fendo.2023.1112363 PubMed PMID: 36824356.
4. Lima JEBF, Moreira NCS, Sakamoto-Hojo ET. Mechanisms underlying the pathophysiology of type 2 diabetes: From risk factors to oxidative stress, metabolic dysfunction, and hyperglycemia. *Mutat Res Toxicol Environ Mutagen*. **2022**;*874–875*:503437. doi:https://doi.org/10.1016/j.mrgentox.2021.503437
5. Khoshjou F, Dadras F. Mitochondrion and its role in diabetic nephropathy. *Iran J Kidney Dis*. **2014**;*8*(5):355–8. PubMed PMID: 25194400.
6. Nolfi-Donegan D, Braganza A, Shiva S. Mitochondrial electron transport chain: Oxidative phosphorylation, oxidant production, and methods of measurement. *Redox Biol.* **2020** Oct;*37*:101674. doi:10.1016/j.redox.2020.101674 PubMed PMID: 32811789.
7. Juan CA, Pérez de la Lastra JM, Plou FJ, Pérez-Lebeña E. The Chemistry of Reactive Oxygen Species (ROS) Revisited: Outlining Their Role in Biological Macromolecules (DNA, Lipids and Proteins) and Induced Pathologies. *Int J Mol Sci*. **2021** Apr;*22*(9). doi:10.3390/ijms22094642 PubMed PMID: 33924958.
8. Nguyen, M. V. C.; Lardy, B.; Rousset, F.; Hazane-Puch, F.; Zhang, L.; Trocmé, C.; et al. Quinone compounds regulate the level of ROS production by the NADPH oxidase Nox4. *Biochemical Pharmacology* **2013**, *85*(11), 1644–1654. doi:https://doi.org/10.1016/j.bcp.2013.03.023.
9. AbuKhader M. Thymoquinone: A promising antidiabetic agent. *Int J Diabetes Dev Ctries*. **2012**;*32*:65–8. doi:10.1007/s13410-012-0067-1
10. Sourris KC, Harcourt BE, Tang PH, Morley AL, Huynh K, Penfold SA, Coughlan MT, Cooper ME, Nguyen TV, Ritchie RH, Forbes JM. Ubiquinone (coenzyme Q10) prevents renal mitochondrial dysfunction in an experimental model of type 2 diabetes. *Free Radic Biol Med*. **2012**;*52*(3):716–23. doi:https://doi.org/10.1016/j.freeradbiomed.2011.11.017

Supplementary Table S2. Barrier-driven formulation strategies for translating quinone pharmacology into diabetes-directed therapy.

Quinone/candidate	Principal developability barrier	Suitable formulation strategy	Intended route or target	Expected pharmaceutical gain	Critical requirement before translation	References
CoQ10	High molecular weight, extreme lipophilicity and oral bioavailability below 5%	Ubiquinol formulations, nanoemulsions, phytosomes, cyclodextrin complexes and TPGS conjugation	Oral systemic delivery; retina, heart and kidney	Improved dissolution, intestinal absorption and tissue exposure	Demonstration that improved exposure produces clinically meaningful metabolic outcomes	[1-3]
PQQ	Hydrophilicity, rapid systemic elimination and limited cellular retention	Liposomes, polymeric carriers or food-matrix incorporation	Oral systemic and mitochondria-directed delivery	Longer circulation and improved intracellular delivery	Exposure–response and renal-clearance characterization	[4, 5]
TQ	Poor water solubility, first-pass metabolism and sensitivity to pH and light	Lipid nanoparticles, nanoemulsions, liposomes and protected polymeric systems	Oral delivery or topical wound application	Increased stability, controlled release and tissue-specific exposure	Standardized loading, photostability, dose and chronic safety	[6-8]
Embelin	BCS class II behaviour and poor aqueous solubility	Liposomes, transfersomes, lipid nanospheres and chitosan nanoparticles	Oral or topical delivery	Improved dissolution, permeability and localized delivery	Comparative safety of metallic versus biodegradable carriers	[9]
Lapachol	Poor absorption and inadequate activity despite high systemic doses	Oil-in-water nanoemulsion or microemulsion	Oral delivery	Sustained release and improved apparent solubility	Proof that enhanced exposure overcomes previous clinical inefficacy	[10-12]
Emodin and related anthraquinones	Poor solubility, rapid glucuronidation/sulfation and chemical degradation	Nanocapsules, lipid carriers and Pluronic F-127 hydrogels	Oral or topical wound delivery	Protection from degradation, reduced first-pass loss and localized release	Control of hepatic and renal toxicity during repeated administration	[13, 14]
Vitamin K derivatives	Variable dietary absorption and	Mixed micelles, emulsions	Oral systemic or wound-	Improved stability, absorption	Monitoring coagulation-related effects	[15, 16]

	acid-related instability	and lipid nanocarriers	directed delivery	and sustained tissue distribution	and drug interactions	
MitoQ/idebenone	Need for controlled mitochondrial accumulation and avoidance of excess redox cycling	Organelle-targeted conjugates or controlled-release systems	Pancreas, kidney, retina and heart	Direct mitochondrial delivery with lower systemic exposure	Definition of the mitochondrial therapeutic window	[17]

References

1. Wang Y, Lilienfeldt N, Hekimi S. Understanding coenzyme Q. *Physiol Rev.* **2024**;*104*(4):1533–610. doi:10.1152/physrev.00040.2023
2. Regulska-Ilow B, Różańska D, Zatońska K, Szuba A. Estimation of Vitamin K Content and Its Sources in the Diet of the Polish Participants of the PURE Study. *Nutrients*. **2022**. doi:10.3390/nu14091917
3. Bus K, Szterk A. Relationship between Structure and Biological Activity of Various Vitamin K Forms. *Foods*. **2021**. doi:10.3390/foods10123136
4. Harris CB, Chowanadisai W, Mishchuk DO, Satre MA, Slupsky CM, Rucker RB. Dietary pyrroloquinoline quinone (PQQ) alters indicators of inflammation and mitochondrial-related metabolism in human subjects. *J Nutr Biochem*. **2013**;*24*(12):2076–84. doi:10.1016/j.jnutbio.2013.07.008
5. Wang D, Wang XH, Yu X, Cao F, Cai X, Chen P, Li M, Feng Y, Li H, Wang X. Pharmacokinetics of anthraquinones from medicinal plants. *Front Pharmacol*. **2021**;*12*:1–19. doi:10.3389/fphar.2021.638993
6. Stielow M, Witczyńska A, Kubryń N, Fijałkowski Ł, Nowaczyk J, Nowaczyk A. The Bioavailability of Drugs—The Current State of Knowledge. *Molecules*. **2023** Dec 11;*28*(24). doi:10.3390/molecules28248038
7. Zhao L, Zheng L. A Review on Bioactive Anthraquinone and Derivatives as the Regulators for ROS. *Mol Basel Switz*. **2023** Dec;*28*(24). doi:10.3390/molecules28248139 PubMed PMID: 38138627.
8. Abdullah, Hussain Y. Anthraquinones and SARS-CoV-2. In: Niaz KBTA of NP in SC 2, editor. Application of Natural Products in SARS-CoV-2. Academic Press; **2023**. p. 171–84. doi:https://doi.org/10.1016/B978-0-323-95047-3.00006-X
9. Halder M, Petsophonsakul P, Akbulut AC, Pavlic A, Bohan F, Anderson E, Maresz K, Kramann R, Schurgers L. Vitamin K: Double Bonds beyond Coagulation Insights into Differences between Vitamin K1 and K2 in Health and Disease. *Int J Mol Sci.* **2019** Feb 19;*20*(4). doi:10.3390/ijms20040896
10. Bank G, Kagan D. Coenzyme Q10: Clinical Update and Bioavailability. Complement Health Pract Rev. **2011** Apr 1;*16*(2):129–37. doi:10.1177/2156587211399438
11. Li Z, Yao R, Guo H, Jing W, Guo X, Liu X, Pan Y, Cao P, Zhang L, Yang J, Cheng X, Wei F. Research Progress on Chemical Compositions, Pharmacological Activities, and Toxicities of Quinone Compounds in Traditional *Chinese Medicines. Toxics*. **2025** Jun;*13*(7). doi:10.3390/toxics13070559 PubMed PMID: 40711004.
12. Siraki AG, Chan TS, O'Brien PJ. Application of Quantitative Structure-Toxicity Relationships for the Comparison of the Cytotoxicity of 14 p-Benzoquinone Congeners in Primary Cultured Rat Hepatocytes Versus PC12 *Cells. Toxicol Sci.* **2004** Sep 1;*81*(1):148–59. doi:10.1093/toxsci/kfh182
13. Dixit VA, Bharatam P V. Toxic Metabolite Formation from Troglitazone (TGZ): New Insights from a DFT Study. *Chem Res Toxicol*. **2011** Jul 18;*24*(7):1113–22. doi:10.1021/tx200110h
14. Tu T, Giblin D, Gross ML. Structural determinant of chemical reactivity and potential health effects of quinones from natural products. *Chem Res Toxicol*. **2011** Sep;*24*(9):1527–39. doi:10.1021/tx200140s PubMed PMID: 21721570.
15. Mtvralashvili D, Abritsova M, Vasilyevykh T, Belous S, Arkhipova O. Melanosis coli as a factor in the development of colorectal cancer. A*mbulatornaya Khirurgiya Ambul Surg Russ*. **2021** Nov 24;*18*(2):84–92. doi:10.21518/1995-1477-2021-18-2-84-92
16. Yao K, Kang Q, Liu W, Chen D, Wang L, Li S. Chronic exposure to tire rubber-derived contaminant 6PPD-quinone impairs sperm quality and induces the damage of reproductive capacity in male mice. *J Hazard Mater*. **2024**;*470*. doi:10.1016/j.jhazmat.2024.134165

17. Rahal A, Kumar A, Singh V, Yadav B, Tiwari R, Chakraborty S, Dhama K. Oxidative stress, prooxidants, and antioxidants: the interplay. *BioMed Res Int*. **2014**;*2014*. doi:10.1155/2014/761264 PubMed PMID: 24587990.
